# An Attention-Based Diffusion Model for Psychometric Analyses

**DOI:** 10.1007/s11336-021-09783-0

**Published:** 2021-07-13

**Authors:** Udo Boehm, Maarten Marsman, Han L. J. van der Maas, Gunter Maris

**Affiliations:** 1 Department of Psychology, University of Amsterdam, Nieuwe Prinsengracht 129B, 1018 WS Amsterdam, The Netherlands; 2 ACT, Iowa City, USA

**Keywords:** diffusion model, response time data, cognitive psychometrics

## Abstract

The emergence of computer-based assessments has made response times, in addition to response accuracies, available as a source of information about test takers’ latent abilities. The development of substantively meaningful accounts of the cognitive process underlying item responses is critical to establishing the validity of psychometric tests. However, existing substantive theories such as the diffusion model have been slow to gain traction due to their unwieldy functional form and regular violations of model assumptions in psychometric contexts. In the present work, we develop an attention-based diffusion model based on process assumptions that are appropriate for psychometric applications. This model is straightforward to analyse using Gibbs sampling and can be readily extended. We demonstrate our model’s good computational and statistical properties in a comparison with two well-established psychometric models.

The advent of computerised testing has made response time (RT), in addition to response accuracy, available as a source of information about the test taker’s latent ability. Psychometricians have generally taken one of two approaches to take advantage of this additional information; whilst some psychometricians treat RTs as a purely statistical entity in their models, others aim to model the cognitive processes underlying the observed RTs in a substantively meaningful way. This latter approach largely relies on sequential sampling models from mathematical psychology (e.g. Ranger & Kuhn, 2018; Rouder et al., 2015; Stone, 1960; Tuerlinckx & De Boeck, 2005; Van der Maas et al., 2011) that were originally developed to account for RT and accuracy data in fast-paced decision and memory tasks (Ratcliff & McKoon, 2008). Although these models have been adopted for psychometric applications with some success, the original emphasis on experimental tasks means that the substantive assumptions in the models are not appropriate for psychometric settings. In the present work, we propose an attention-based diffusion model, which we derive from cognitive process assumptions that are more appropriate in the psychometric setting of performance tests.

Diffusion-type models conceptualise the process by which a test taker chooses between two response options, A and B, as the noisy accumulation of information. In their most basic mathematical form, diffusion-type models include five parameters, $t_0$, *v*, *s*, *a*, and *z* (Fig. 1). These parameters account for the joint distribution of response time $t \in \mathbb {R}^+$ and choice $x \in \{0,1\}$, where $x=1$ corresponds to choosing option A and $x=0$ corresponds to choosing option B.

For applications to experimental data, Ratcliff (1978) proposed a particular set of substantive interpretations for the model parameters that, together with the mathematical form of the model, gives rise to his diffusion model (DM). The DM decomposes response time as $t = t_0 + t_d$. The constant $t_0$ is the non-decision time, which accounts for the time spent on cognitive processes not related to the choice process, such as visual encoding and the execution of a motor response. The distribution of the decision time $t_d$ is determined by the remaining four model parameters. The DM represents the choice process as a repeated sampling process where the decision maker samples and integrates noisy information about the two response options from a normal distribution with mean given by the drift rate parameter *v* and standard deviation given by the diffusion coefficient *s*. The diffusion coefficient, which represents the within-trial volatility of the information being sampled, is assigned a fixed value, typically 0.1 or 1. The drift rate, which represents the rate at which the decision maker processes information, is estimated from the observed data. Each response option is associated with a decision threshold, one located at 0 and the other located at *a*, and *a* is referred to as the boundary separation. Boundary separation represents the response caution the decision maker applies in a given decision problem, and this model parameter is estimated from the observed data. Finally, the sampling process starts at the point *z*, located between the two decision thresholds, and continues until a decision threshold is reached. The starting point represents the decision maker’s a priori preference for one response option over the other and is again estimated from the data. The final decision time $t_d$ is determined by the point where the accumulated evidence first crosses a decision threshold, upon which the test taker commits to the corresponding response option.

**Fig. 1:**
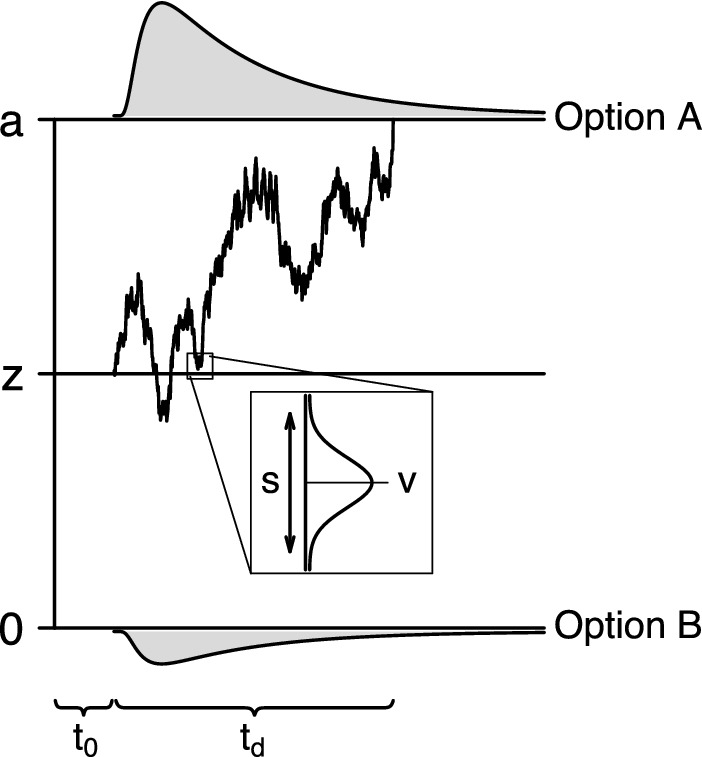
Structure of diffusion-type models. Distributions of decision times for option A and option B are shown above and below the corresponding decision boundary.

Implicit in the DM’s interpretation of the model parameters are four assumptions that might be plausible in an experimental context but appear less appropriate for data from a performance test. These assumptions are the independence of decision and non-decision processes, a fixed diffusion coefficient, independence of decision boundaries and diffusion coefficient, and biases in the starting point of the decision process. Concerns about the applicability of the DM outside fast-paced experimental tasks were also expressed by Ratcliff and McKoon (2008), who warned that the DM was developed for task settings that involve only a single processing stage, and is not suited to tasks that elicit longer RTs (see also Lerche & Voss, 2019; Ratcliff & Frank, 2012; Ratcliff et al., 2004). To address these inadequacies of the DM, we propose four cognitive process assumptions that are appropriate for the analysis of performance test data. These assumptions in conjunction with the standard mathematical form of diffusion-type models constitute our Attention-Based Diffusion Model (ABDM).

Firstly, the additive decomposition $t = t_0 + t_d$ in the DM embodies the assumption that decision and non-decision processes are completely independent; the duration and number of non-decision processes affect neither the duration nor the outcome of the decision process. This assumption might be plausible in simple experimental tasks that involve only a single processing stage, where non-decision processes can be reasonably assumed not to interrupt the decision process. However, in more complex cognitive tasks that require multiple processing states, non-decision process might occur at the transition between different processing stages and can interrupt ongoing cognitive processes. Hence, the duration and number of non-decision processes in complex tasks are related to the duration and outcome of the decision process. A prime example of an interruptive non-decision process is mind wandering, the diversion of attentional resources away from an ongoing cognitive task to task-unrelated thoughts (Schooler et al., 2014). Several studies have found that a higher frequency of mind-wandering is correlated with worse outcomes on tests of cognitive performance such as sustained attention (Allan Cheyne et al., 2009), working memory, and general intelligence (Mrazek et al., 2012; see Mooneyham & Schooler, 2013, for a review). In the context of the DM, Hawkins et al. (2015) argue that mind-wandering is associated with increased variability in drift rate across experimental trials. An association between drift rate and non-decision processes is further supported by Voss et al. (2004) finding that an experimental manipulation that increased the duration of non-decision processes also resulted in a lower drift rate.

In our ABDM, we assume that lapses in attention directly affect the decision process. Rather than additively decomposing RTs, we assume that non-decision processes lead to a decrease in the rate of information processing. In more formal terms, this assumption can be understood via the random walk approximation of Brownian motion (see Appendix A). The classic DM is the limit of a random walk where, at each time step, the decision maker processes information that favours response option *A* with probability $p_A$ and processes information that favours response option *B* with probability $p_B=1-p_A$. The time spent on non-decision processes is then accommodated by adding a constant $t_0$ to the decision time $t_d$. Our ABDM, on the other hand, is the limit of a random walk where, at each time step, the decision maker processes information that favours response option *A* with probability $p_A$, processes information that favours response option *B* with probability $p_B$, and engages in non-decision processes with probability $1-(p_A+p_B)$. The effect of non-decision processes is manifested in a lower drift rate *v* and thus becomes an integral part of the decision time. Although non-interruptive non-decision processes might be present, their contribution to the observed RT is negligible. We will return to this point in our simulation studies below.

Secondly, setting the diffusion coefficient *s* to a fixed value in the DM embodies the assumption that the volatility of the information being accumulated is constant across experimental trials and conditions. Setting the diffusion coefficient to a constant value is often justified by the need to fix one of the DM parameters to identify the model. However, as pointed out by Donkin et al. (2009), most applications of the DM make additional assumptions, such as equality of boundary settings across experimental trials and conditions, that in themselves suffice to identify the model. Hence, a fixed diffusion coefficient tacitly introduces the additional assumption that information accumulation is equally volatile throughout the experiment. This assumption might often be plausible in experimental settings where stimulus materials are simple and homogeneous across trials, and researchers have full control over all statistical properties of the stimuli. In the popular random dot motion task, for instance, stimuli consist of a cloud of pseudo-randomly moving dots that is constructed to elicit the same level of visual noise across experimental trials (Britten et al., 1992). Researchers can control the difficulty of the experimental task by increasing or decreasing the proportion of consistently moving dots independent of the level of visual noise. However, in psychometric settings stimulus materials are complex and vary across items, and researchers have limited control over the statistical properties of the stimuli. On a reading test, for instance, words will differ in length and shape between items, which will introduce uncontrolled visual noise. To accommodate this natural item-specific within-trial volatility in our ABDM, we allow the diffusion coefficient *s* to vary across items.

Thirdly, the independence of boundary separation *a* and diffusion coefficient *s* in the DM suggests that decision makers adjust their response caution irrespective of the volatility of the information that becomes available. Although this assumption has been a default in applications of the DM to experimental data, a recent debate in the literature has highlighted the controversial nature of this assumption. Several authors have suggested that decision makers adjust their boundary separation in response to changes in information volatility (e.g. Deneve, 2012; Ditterich, 2006; Shadlen & Kiani, 2013). Recent experimental results suggest that such adjustments can even occur within a single decision, with decision makers lowering their response caution in the face of lower signal-to-noise ratios (e.g. Drugowitsch et al., 2012; Hanks et al., 2011; Thura et al., 2012).

In our ABDM, we assume that decision makers accommodate changes in information volatility across items by adjusting their response caution. Several authors have suggested different functional relationships between volatility and response caution (e.g. Drugowitsch et al., 2012; Hanks et al., 2011; Thura et al., 2012). Here, we assume that the decision maker’s response caution is a fixed ratio of the volatility, that is, ${s}/{a}=c$. This assumption corresponds to linearly decreasing decision boundaries with increasing volatility in the classic DM and yields a particularly simple expression for the likelihood of our ABDM.

Finally, in the DM the starting point of the sampling process *z* is assumed unknown and is estimated from the observed data. This assumption is reasonable in experimental settings that manipulate the prior probability of one response option being correct or receiving a reward; the strength of the a priori preference for a response option induced by the manipulation might vary across decision makers and therefore needs to be estimated. However, in the absence of such manipulations the sampling process is regularly found to be unbiased (e.g. Ratcliff et al., 2004; Voss et al., 2004; Wagenmakers et al., 2008; see also Matzke and Wagenmakers’s (2009) review of DM parameter values found in empirical studies). In psychometric applications to performance tests, unbiasedness of the sampling process even becomes a necessity. An a priori preference for one response option over another is indicative of a flawed test construction, and unbiasedness is assumed by default in psychometric models (Haladyna, 2004; Osterlind, 1998). Therefore, we assume in our ABDM that the psychometric test does not induce a response bias, that is, the starting point of the accumulation process is in the middle of the decision boundaries, $z={a}/{2}$.

Taken together, our ABDM assumes that decision makers choose between two response options by accumulating noisy information about the options. Lapses in attention that interrupt the accumulation process decrease the rate of information accumulation. The volatility of the available information depends on the item, which decision makers accommodate by lowering their response caution in proportion to the volatility. Moreover, assuming proper test construction, decision makers exhibit no a priori preference for one response option over the other. A further theoretical exploration of the relationship between the DM and our ABDM can be found in Appendix B.

These assumptions yield a model with three parameters, namely the rate of information accumulation *v*, the volatility parameter *s* and the ratio of response caution and volatility *c*. The values of these parameters might vary across persons and items. The rate parameter *v* is clearly subject to person and item effects as the rate at which person *p*, $p = 1,\ldots N_P$, answering item *i*, $i = 1, \ldots , N_I$, depends on the person’s ability as well as the difficulty of the item. Following Tuerlinckx and De Boeck (2005), we assume additive effects of the person’s ability $\theta _p$ and the item easiness $\beta _i$ on *v*:

$$\begin{aligned} v_{ip} = \beta _i + \theta _p. \end{aligned}$$

As pointed out above, information volatility varies across items, which is expressed in the item effect $\alpha _i$ on *s*:

$$\begin{aligned} s_{ip} = \alpha _i. \end{aligned}$$

As we will see in the following, having additive person and item effects on *s* would yield an unidentified model. Although identification of the model could also be achieved with a different random and fixed effects structure for $v_{ip}$ and $s_{ip}$, our choice here yields a 2-PLM (Birnbaum, 1968) for the probability of a correct response.

Finally, the volatility-to-response caution ratio *c* represents the adjustment of a decision maker’s response caution to the information volatility of an item, and *c* is therefore constant across items but might differ across decision makers. Here, we will follow the approach recently advocated in neuroscience (Cisek et al., 2009; Shadlen & Kiani, 2013; Thura et al., 2012; Thura & Cisek, 2014; Thura et al., 2014), where the volatility-to-caution ratio is fixed to the same value for all decision makers. This approach conveys considerable computational advantages, as we discuss in the following.

The joint density of the response time *t* and choice *x* in diffusion-type models is given by (Ratcliff, 1978):

1 $$\begin{aligned} f(t, x) =&\, \frac{\pi s^2}{a^2} \exp {\left( [ax-z]\frac{v}{s^2}-t\frac{v^2}{2s^2} \right) } \nonumber \\&\times \sum _{k=1}^{\infty }\left[ k\sin \left( \frac{\pi k [ax-2xz+z]}{a}\right) \exp {\left( -\frac{\pi ^2 s^2 k^2}{2a^2} t\right) }\right] . \end{aligned}$$

From this density, expressions can be derived for the mean RT (Cox & Miller, 1970; Laming, 1973):

2 $$\begin{aligned} \mathbb {E}(T) = \frac{a}{2v} \frac{1-\exp {\left( -\frac{va}{s^2}\right) }}{1 + \exp {\left( -\frac{va}{s^2}\right) }}, \end{aligned}$$

and the probability of a correct response:

3 $$\begin{aligned} \mathbb {P}(X=1) = \frac{\exp {\left( -\frac{2zv}{s^2}\right) }-1}{\exp {\left( -\frac{2av}{s^2}\right) }-1}. \end{aligned}$$

The substantive assumptions derived above yield for the density of our ABDM:

4 $$\begin{aligned} f(t, x) = \pi c^2 \exp {\left( [x-1/2]\frac{v}{cs}-t\frac{v^2}{2s^2} \right) } \times \sum _{k=1}^{\infty }\left[ k\sin \left( \frac{\pi k}{2}\right) \exp {\left( -\frac{\pi ^2 c^2 k^2}{2} t\right) }\right] . \end{aligned}$$

The form of the model density (4) shows that additive person and item effects on *s* would lead to an unidentifiable model because *v* and *s* always appear in a ratio where person and item effects on the two parameters cancel. Our substantive assumptions mean that the probability of a correct response given in Eq. (3) becomes:

5 $$\begin{aligned} \mathbb {P}(X=1) = \frac{1}{1+\exp {\left( -\frac{v_{ip}}{cs_{i}}\right) }}, \end{aligned}$$

which is a 2-PLM (see also Tuerlinckx & De Boeck, 2005).

Finally, the volatility-to-caution ratio *c* is the only model parameter that appears in the series expression in Eq. (4), whilst *s* and *v* only appear in the exponential term. As *c* is assumed to be constant across persons and items, the estimation of the person and item effects on *s* and *v* can be separated from the estimation of *c*. This conveys a considerable computational advantage as the costly evaluation of the series expression is limited to the estimation of *c*. In the next section, we will first introduce a Bayesian implementation of our ABDM that enables efficient estimation of the person and item effects on *s* and *v*, which we will subsequently use to estimate *c*.

## Bayesian Implementation

Our ABDM lends itself naturally to Bayesian analysis due its functional form. Specifically, assuming the parameterisation introduced above, where the rate of information accumulation for person *p* answering item *i* is determined by the person’s ability $\theta _p$ and the item easiness $\beta _i$, $v_{ip} = \beta _i + \theta _p$, and the volatility varies across items, $s_i = \alpha _i$, the density of the ABDM is:

6 $$\begin{aligned} f(t_{ip},x_{ip} \mid \beta _i, \theta _p, \alpha _i) =&\, \pi c^2 \exp {\left( [x_{ip}-1/2]\frac{\beta _i + \theta _p}{c \alpha _i}-t_{ip}\frac{(\beta _i + \theta _p)^2}{2\alpha _i^2} \right) } \nonumber \\&\times \sum _{k=1}^{\infty }\left[ k\sin \left( \frac{\pi k }{2} \right) \exp {\left( -\frac{\pi ^2 c^2 k^2}{2} t_{ip}\right) }\right] \end{aligned}$$

and dropping all constants not related to $\beta _i$, $\theta _p$, or $\alpha _i$ and letting $\tilde{\alpha }_i=\alpha ^{-1}_i$:

$$\begin{aligned} \propto \exp { \left( [x_{ip}-1/2]\tilde{\alpha }_i\frac{(\beta _i + \theta _p)}{c}-t_{ip}\tilde{\alpha }^2_i\frac{(\beta _i + \theta _p)^2}{2} \right) }. \end{aligned}$$

If we assume for now that the value of *c* is known, then this form of the density suggests a conjugate analysis for the estimation of the remaining parameters. Choosing a multivariate normal distribution as joint prior for the model parameters $\beta _i$, $\theta _p$, and $\tilde{\alpha }_i$, and an inverse Wishart prior for the variance–covariance matrix, will yield a multivariate normal posterior distribution for all parameters. Due to the interpretation of the volatility $\tilde{\alpha }_i$ as a standard deviation, only positive values are admissible for this parameter and the prior distribution for $\tilde{\alpha }_i$ needs to be truncated at zero, which complicates the form of the joint posterior distribution considerably. Nevertheless, conjugacy will still hold for the full conditional posterior distributions of $\beta _i$, $\theta _p$ and $\tilde{\alpha }_i$. Hence, our model can be straightforwardly analysed using Gibbs sampling.

### Random Effects

The likelihood function of our ABDM, Eq. (6), has a simple form that makes it amenable to extension. The terms of the series expression do not depend on any of the model parameters and can therefore be dropped in analyses of the likelihood function, which leaves a simple exponential family model. Moreover, the item and person effects on drift rate, $\beta _i$ and $\theta _p$, and the item effects on the information volatility, $\tilde{\alpha }_i$, only appear in the first and second powers. Hence, the full-conditional distributions we introduce below take the form of a normal kernel, which allows for simple conjugate analysis and easy sampling-based estimation.

One problem that is regularly of interest in psychometrics is the modelling of unobserved heterogeneity. We show here how our ABDM can be extended to accommodate a random effects structure. Extensions to other statistical problems such as missing data, inclusion of manifest predictor variables, or the addition of autoregressive components are straightforward.

To model heterogeneity in the data, we assume the following population-level distributions:

$$\begin{aligned} \begin{array}{lll} \beta _i \sim \mathscr {N}(\mu _I, \sigma ^2_I),&\theta _p \sim \mathscr {N}(\mu _P, \sigma ^2_P),&\tilde{\alpha }_i \sim \mathscr {N}(\mu _A, \gamma ^2_A)[0, \infty ). \end{array} \end{aligned}$$

Here, $\mathscr {N}(\mu ,\sigma ^2)$ denotes the normal distribution with mean $\mu$ and variance $\sigma ^2$. The expression $[0, \infty )$ indicates the support of the truncated distribution. The population-level parameters can be assigned prior distributions in a similar manner:

$$\begin{aligned} \begin{array}{llll} \mu _P \sim \mathscr {N}(\nu _{P0}, \tau ^2_{P0}), &{} \sigma ^2_P \sim invG(a_{P0}, b_{P0}), &{} \mu _I \sim \mathscr {N}(\nu _{I0}, \tau ^2_{I0}), &{} \sigma ^2_I \sim invG(a_{I0}, b_{I0}).\\ \mu _A \sim \mathscr {N}(\nu _{A0}, \tau ^2_{A0}), &{} \gamma ^2_A \sim invG(a_{A0}, b_{A0}). &{} &{} \end{array} \end{aligned}$$

Here, *invG*(*a*, *b*) denotes the inverse gamma distribution with shape parameter *a* and scale parameter *b*.

The full-conditional conjugate posterior distributions for the person and item effects on drift rate $v_{ip}$ for $N_P$ persons answering $N_I$ items are:

$$\begin{aligned} \begin{array}{ll} \beta _i \sim \mathscr {N}(\hat{\beta }_{i,N_P}, \hat{\sigma }^2_{i,N_P}),&\theta _p \sim \mathscr {N}(\hat{\theta }_{p,N_I}, \hat{\sigma }^2_{p,N_I}), \end{array} \end{aligned}$$

where

$$\begin{aligned} \begin{array}{ll} \hat{\sigma }^2_{i,N_P} = \left( \sum _{p=1}^{N_P} \frac{ t_{ip}}{\alpha _i^2} + \frac{1}{\sigma ^2_I} \right) ^{-1}, &{} \hat{\beta }_{i,N_P} = \left( \sum _{p=1}^{N_P}\frac{ (x_{ip}-1/2)}{c \alpha _i} - \sum _{p=1}^{N_P}\frac{ t_{ip} \theta _p}{\alpha _i^2} + \frac{\mu _{I}}{\sigma ^2_I} \right) \hat{\sigma }^2_{i,N_P} \\ \hat{\sigma }^2_{p,N_I} = \left( \sum _{i=1}^{N_I}\frac{ t_{ip}}{\alpha _i^2} + \frac{1}{\sigma ^2_P} \right) ^{-1}, &{} \hat{\theta }_{p,N_I} = \left( \sum _{i=1}^{N_I} \frac{ (x_{ip}-1/2)}{c \alpha _i} - \sum _{i=1}^{N_I}\frac{ t_{ip} \beta _i}{\alpha _i^2} + \frac{\mu _{P}}{\sigma ^2_P} \right) \hat{\sigma }^2_{p,N_I}. \end{array} \end{aligned}$$

Similarly, the full-conditional conjugate posterior distribution for item effects on the reciprocal volatility parameter $s^{-1}_{ip}$ is:

$$\begin{aligned} \tilde{\alpha }_i \sim \mathscr {N}(\hat{\alpha }_{i,N_P}, \hat{\gamma }^2_{i,N_P})[0, \infty ), \end{aligned}$$

where

$$\begin{aligned} \begin{array}{ll} &{} \hat{\gamma }^2_{i,N_P} = \gamma ^2_A \left( \sum _{p=1}^{N_P} t_{ip}(\beta _i+\theta _p)^2\gamma ^2_A + 1 \right) ^{-1},\\ &{} \hat{\alpha }_{i,N_P} = \left( \mu _{A}/\gamma ^2_{A} + \sum _{p=1}^{N_P} (x_{ip}-1/2)(\beta _i+\theta _p)/c \right) \hat{\gamma }^2_{i,N_P}. \end{array} \end{aligned}$$

The full-conditional posterior distributions for the population-level parameters are:

$$\begin{aligned} \begin{array}{llll} \mu _P &{} \sim \mathscr {N}(\nu _{P,N_P}, \tau ^2_{P,N_P}), &{} \text { where } \nu _{P,N_P} = \frac{\tau ^2_P \sum _{p=1}^{N_P} \theta _p + \sigma ^2_P \nu _{P,0}}{\tau ^2_P N_P + \sigma ^2_P}, &{} \tau ^2_{P,N_P} = \frac{\tau ^2_P \sigma ^2_P}{\tau ^2_P N_P + \sigma ^2_P}\\ \sigma ^2_P &{} \sim invG(a_{P,N_P}, b_{P,N_P}), &{} \text { where } a_{P,N_P} = a_{P,0} + N_P/2, \\ &{} b_{P,N_P} = \sum _{p=1}^{N_P} (\theta _p - \mu _P)^2/2 + b_{P,0}\\ \mu _I &{} \sim \mathscr {N}(\nu _{I,N_I}, \tau ^2_{I,N_I}), &{} \text { where } \nu _{I,N_I} = \frac{\tau ^2_I \sum _{i=1}^{N_I} \beta _i + \sigma ^2_I \nu _{I,0}}{\tau ^2_I N_I + \sigma ^2_I}, &{} \tau ^2_{I,N_I} = \frac{\tau ^2_I \sigma ^2_I}{\tau ^2_I N_I + \sigma ^2_I}\\ \sigma ^2_I &{} \sim invG(a_{I,N_I}, b_{I,N_I}), &{} \text { where } a_{I,N_I} = a_{I,0} + N_I/2, \\ &{} b_{I,N_I} = \sum _{i=1}^{N_I} (\beta _i - \mu _I)^2/2 + b_{I,0}\\ \mu _A &{} \sim \mathscr {N}(\nu _{A,N_I}, \tau ^2_{A,N_I}), &{} \text { where } \nu _{A,N_I} = \frac{\tau ^2_A \sum _{i=1}^{N_I} \tilde{\alpha }_i + \gamma ^2_A \nu _{A,0}}{\tau ^2_A N_I + \gamma ^2_A}, &{} \tau ^2_{A,N_I} = \frac{\tau ^2_A \gamma ^2_A}{\tau ^2_A N_I + \gamma ^2_A}\\ \gamma ^2_A &{} \sim invG(a_{A,N_I}, b_{A,N_I}), &{} \text { where } a_{A,N_I} = a_{A,0} + N_I/2,\\ &{} b_{A,N_I} = \sum _{i=1}^{N_I} (\tilde{\alpha }_i - \mu _A)^2/2 + b_{A,0}. \end{array} \end{aligned}$$

Let $\vec {t}=[t_{11}, \ldots , t_{IP}]$ be the vector of response times and let $\vec {x}=[x_{11}, \ldots , x_{IP}]$ be the vector of accuracies. Moreover, denote by $\Theta$ the set of all item, person, and population-level parameters, then:

$$\begin{aligned}&f(\vec {t},\vec {x} \mid \Theta )f(\Theta ) \propto \exp {\left( \sum _{i=1}^{N_I} \sum _{p=1}^{N_P} \left( \tilde{\alpha }_i[x_{ip}-1/2]\frac{\beta _i + \theta _p}{c}-t_{ip}\tilde{\alpha }^2_i\frac{(\beta _i + \theta _p)^2}{2} \right) \right) }\\&\quad \times \exp {\left( -\left( \sum _{i=1}^{N_I} \frac{(\beta _i - \mu _{I})^2}{2\sigma ^2_I} + \sum _{p=1}^{N_P} \frac{(\theta _p - \mu _{P})^2}{2\sigma ^2_P} + \sum _{i=1}^{N_I} \frac{(\tilde{\alpha }_i - \mu _{A})^2}{2\gamma ^2_A}\right) \right) }\\&\quad \times \exp {\left( -\left( \frac{(\mu _{I} - \nu _{I0})^2}{2\tau ^2_{I0}} + \frac{(\mu _{P} - \nu _{P0})^2}{2\tau ^2_{P0}} + \frac{(\mu _{A} - \nu _{A0})^2}{2\tau ^2_{A0}} + \frac{b_I}{\sigma ^2_I} + \frac{b_P}{\sigma ^2_P} + \frac{b_A}{\gamma ^2_A} \right) \right) } \\&\quad \times \sigma ^{-2(a_I+1+N_I/2)}_{I} \sigma ^{-2(a_P+1+N_P/2)}_{P} \gamma ^{-2(a_A+1+N_I/2)}_{A}. \end{aligned}$$

This random effects extension of our model is not identified, which we address by fixing two model parameters:

$$\begin{aligned} \mu _P = 0, \quad \sigma ^2_P = 1. \end{aligned}$$

The first constraint addresses the trade-off between the $\beta _i$ and $\theta _p$, where adding a constant to all $\beta _i$ and subtracting the same constant from all $\theta _p$ yields the same value for the likelihood as the original values $\beta _i$ and $\theta _p$. By requiring $\mu _P = 0$, we fix the location of the person effects on drift rate. The second constraint addresses the indeterminate scale of the item and person effects, where multiplying all $\beta _i$ and $\theta _p$ by a constant is compensated for by multiplying the corresponding variance terms by the same constant.

### Regression Models for Person Effects

Our ABDM allows for the inclusion of regression models for the latent person effects and can also be extended to latent regression of item parameters on manifest covariates. We will illustrate this for the case where the person effects $\theta _p$ are regressed on a set of covariates. A more complete discussion of latent regression models can be found in, for example, Fox (2001), Fox and Glas (2006), Mislevy (1985), and Zwinderman (1991).

Due to the functional form of our ABDM, the addition of a regression extension is straightforward. The random effects structure for the latent variable of interest can be replaced by a regression equation. For an appropriately chosen prior distribution on the regression coefficients and residual variance, this yields normal–inverse gamma posterior distributions for the regression coefficients and residual variance and leaves the normal–normal conjugacy for the latent variables intact.

To regress the person effect $\theta _p$ on a set of covariates, we specify the regression model as:

7 $$\begin{aligned} \theta _p = \vec {\lambda }^T \vec {y}_p + \epsilon _p, \quad \epsilon _p \sim \mathscr {N}(0, \sigma ^2_{res}), \end{aligned}$$

where $\vec {y}_p$ is a $(K+1) \times 1$ vector with first entry equal to 1 and the remaining *K* entries being the manifest covariate values for person *p*, $\vec {\lambda }$ is a $(K+1) \times 1$ vector of regression weights, and $\epsilon _p$ is a normally distributed error term with mean 0 and variance $\sigma ^2_{res}$. If we assign the non-informative prior distribution $[\vec {\lambda },\sigma ^2_{res}] \propto 1/\sigma ^2_{res}$ and replace the random effects term for $\theta _p$ by the regression model in Eq. (7), our model becomes:

$$\begin{aligned} f(\vec {t},\vec {x} \mid \vec {\theta }, \vec {\lambda }, \sigma ^2_{res})&f(\vec {\lambda })f(\sigma ^2_{res}) \propto \exp \left( -\sum _{p=1}^{N_P}\theta ^2_p \left( \sum _{i=1}^{N_I} \frac{t_{ip}\tilde{\alpha }^2_i}{2} + \frac{1}{2\sigma ^2_{res}}\right) \right) \\&\times \exp \left( 2\sum _{p=1}^{N_P} \theta _p \left( \sum _{i=1}^{N_I} \tilde{\alpha }_i\frac{[x_{ip}-1/2]}{2c} - \sum _{i=1}^{N_I} t_{ip}\tilde{\alpha }^2_i\frac{\beta _i}{2} + \frac{\vec {\lambda }^T \vec {y}_p}{2 \sigma ^2_{res}} \right) \right) \\&\times (\sigma ^2_{res})^{-\left( N_p/2+1\right) }. \end{aligned}$$

Here, $\vec {\theta }$ denotes the $N_p \times 1$ vector of person effects on drift rate. Note that replacing the expression for the random effect on $\theta _p$ by the regression model replaces the variance term $\sigma ^2_P$ by the residual variance $\sigma ^2_{res}$. From the expression above, it can be seen that the full-conditional posterior distributions for $\vec {\lambda }$, $\sigma ^2_{res}$, and $\theta _p$ have the form (for details on the derivation, see chapter 14 in Gelman et al., 2013):

$$\begin{aligned} \begin{array}{lll} \vec {\lambda } \sim \mathbf {\mathscr {N}}(\hat{\lambda }, V_{\lambda }\sigma ^2_{res}),&\sigma ^2_{res} \sim invG(\frac{n}{2}, \frac{n-K}{2}\hat{\sigma }^2_{res}),&\theta _p \sim \mathscr {N}(\hat{\theta }_{p,N_I}, \hat{\sigma }^2_{p,N_I}), \end{array} \end{aligned}$$

whereHere, *Y* is the $N_p \times (K+1)$ design matrix with all entries of the first column equal to 1 and the remaining columns being the *K* vectors containing the values of the predictors $Y_1,\ldots ,Y_K$. Hence, MCMC samples for latent regression in the ABDM can be easily obtained using Gibbs-sampling.

As before, this extended model is unidentified, which we address by fixing two parameters. Firstly, we set $\lambda _0=0$ to fix the location of the person effects $\theta _p$, which we previously addressed by setting $\mu _P=0$. Secondly, we set $\sigma ^2_{res} = 1$ to fix the scale of the person effects $\theta _p$, which we previously addressed by setting $\sigma ^2_P=1$.

### MCMC Sampling

The analysis of our model relies on MCMC sampling from the posterior distribution. The full-conditional posterior distributions for all parameters are conjugate to the prior distributions and have standard functional forms. We can therefore easily obtain MCMC samples using the Gibbs sampler (Gelfand & Smith, 1990), which is implemented by alternately sampling from the full-conditional posterior distributions. Custom-written R code that implements the Gibbs sampler for our ABDM is available on the project’s OSF page https://osf.io/fh4wp/.

### Estimation of the Volatility-to-Caution Ratio

As pointed out above, estimation of the volatility-to-caution ratio *c* requires the evaluation of the full density of the ABDM, including the series expression in Eq. (6). The assumption that *c* is constant across persons and items means that the estimation problem is one dimensional. Fully Bayesian estimation of *c* would be computationally intractable. However, simple iterative optimisation can be carried out by estimating the person and item effects $\beta _i$, $\theta _p$, and $\tilde{\alpha }_i$ for each candidate value $\tilde{c}$ using Gibbs sampling and using the ABDM likelihood in Eq. (6) evaluated at the posterior means as the objective function.

If the objective function has a unique global maximum, efficient adaptive estimation can be achieved by the golden section algorithm (Kiefer, 1953). If the objective function does not have a unique global maximum, other approaches such as a grid search or adaptive gradient descent methods can be employed. Initial values for the latter type of search algorithm can be obtained from the moment estimator of *c*. Specifically, if we fix all item and person effects on the ABDM parameters and write $p=\mathbb {P}(X=1)$ for the probability of a correct response, then Eq. (3) gives:

$$\begin{aligned} p = \frac{1}{1+\exp {\left( -\frac{v}{cs}\right) }} \iff \frac{v}{cs} = \log \left( \frac{p}{1-p} \right) {=:} L. \end{aligned}$$

Writing *M* for the mean RT and substituting *L* into the expression in Eq. (2) gives:

$$\begin{aligned} M = \frac{a}{2v} \frac{1-\exp {\left( -\frac{va}{s^2}\right) }}{1 + \exp {\left( -\frac{va}{s^2}\right) }} = \frac{1}{2Lc^2}\frac{1-\exp {\left( -L\right) }}{1+\exp {\left( -L\right) }}. \end{aligned}$$

Solving for *c* and using the fact that $c>0$, we get the estimator:

$$\begin{aligned} \hat{c} = \sqrt{\frac{1}{2LM}\frac{1-\exp {\left( -L\right) }}{1+\exp {\left( -L\right) }}}. \end{aligned}$$

As the joint likelihood of the data conditional on *c* had a unique global maximum in all our example applications, we used a golden section algorithm to estimate *c*.

## Simulation Study

**Fig. 2:**
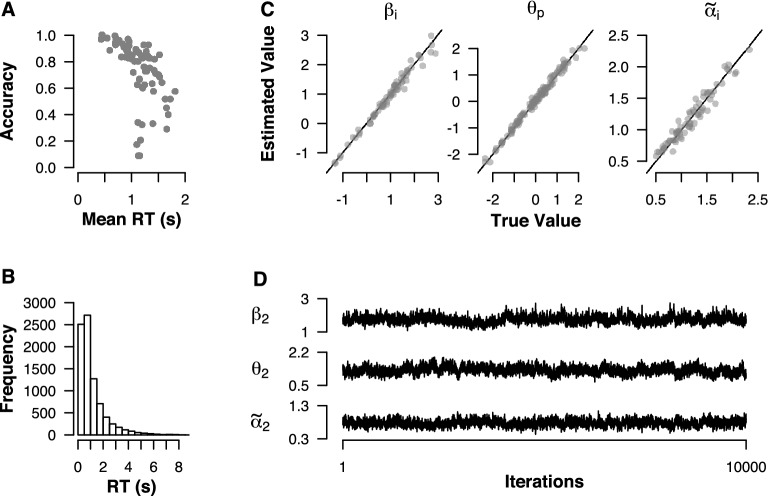
Simulation study with 120 persons answering 70 items. **a** Accuracy and mean RT for simulated items. **b** Marginal RT distribution over items and persons. **c** Estimated and true values for the item ($\beta _i$) and person ($\theta _p$) effects on the rate or information accumulation, and for the item effects on the reciprocal volatility ($\tilde{\alpha }_i$). **d** Example MCMC chains for item and person effects after a burn-in period of 2,000 samples.

We tested the sampling behaviour of our ABDM in a simulation study. We used the rdiffusion function from the RTdists R package (Singmann et al., 2016) to generate RT and choice data for 120 persons answering 70 items. The generating population-level parameters were set to $\mu _I=1$, $\sigma ^2_I=1$, $\mu _P=0$, $\sigma ^2_P=1$, $\mu _{A}=1$, and $\gamma ^2_{A}={1}/{2}$. We truncated the generating distribution for $\tilde{\alpha }_i$ at ${1}/{2}$ instead of 0 to avoid unrealistically large values of the information volatility in the simulated data. We furthermore set the caution-to-volatility ratio to $c = 0.35$. Panel A of Fig. 2 shows the joint distribution of mean accuracies and mean RTs for the simulated items. As can be seen, both variables spanned a realistic range of values. Panel B of Fig. 2 shows the marginal distribution of RTs. All RTs fell in the range below 10s. Moreover, the distribution exhibits the skew and long tail that is characteristic of RT data.

We implemented our random effects model using custom-written R code in combination with the algorithm for sampling from truncated normal distributions implemented in the truncnorm package (Mersmann et al., 2018). For the estimation of the volatility-to-caution ratio *c*, we used the method described in the preceding section. R code for the simulations and model fitting is available on OSF: https://osf.io/fh4wp/. We considered the search space $C=[0.1,1]$. We set the maximum number of iterations for the golden section algorithm to 10, and we terminated the search if the relative change in the log-likelihood between iterations was less than 1%. To estimate the person and item parameters for each candidate value of *c*, we generated a single chain of 2,000 MCMC samples and discarded the first 1,000 samples as burn-in samples. Once we had obtained an estimate of *c*, we generated the final set of three chains with 12000 MCMC samples. We discarded the first 2000 samples as burn-in samples. The golden section algorithm yielded an estimate of $\hat{c}=0.343$. Panel C of Fig. 2 shows the posterior mean estimates of the person and item effects $\theta _p$, $\beta _i$, and $\tilde{\alpha }_i$. As can be seen, all parameters could be recovered accurately. Panel D of Fig. 2 shows three example chains for the person and item effects. All three parameters, $\beta _2$, $\theta _2$, and $\tilde{\alpha }_2$, showed good sampling behaviour with relatively small autocorrelations.

Table 1 shows a summary of the potential scale reduction factor $\hat{R}$ (Gelman & Rubin, 1992). As can be seen, $\hat{R}$ was below 1.02 for all population parameters. Moreover, $\hat{R}$ did not exceed 1.03 for all chains for all parameters $\beta _i$, $\theta _p$, and $\tilde{\alpha }_i$. Taken together, the results of our simulation study show that person and item effects on drift rate and item effects on the diffusion coefficient can be estimated with good efficiency and generating values can be recovered with high accuracy.

**Table 1:** Convergence results for simulated data.

Population parameter	$\hat{R}$	Person/item parameter	$\hat{R}$ Quantiles		
$\mu _I$	1.012		0.25	0.5	0.75
$\mu _A$	1.004	$\beta _i$	1.009	1.015	1.021
$\sigma ^2_I$	1.002	$\theta _p$	1.014	1.017	1.018
$\gamma ^2_A$	1.002	$\tilde{\alpha }_i$	1.002	1.002	1.003

Quantiles are computed across persons or items.

In a further set of simulations, we explored the effect of a neglected additive non-decision time on the ABDM parameter estimates. As pointed in the introduction, our ABDM assumes that non-decision processes affect the decision time by interrupting decision processes, and changes in non-decision time are therefore reflected by changes in the rate of information processing. However, it might be argued that some quick-paced non-decision processes such as stimulus encoding and response execution do not affect decision processes and result in a small additive non-decision component of the observable response time. To gauge the effect of such a small additive non-decision component on parameter estimates in our ABDM, we generated data from the ABDM and added a small non-decision component to the generated RTs.

As in our first simulation, we generated RT and accuracy data for 120 persons answering 70 items with the same settings for the generating population parameters. We generated data with four different values for the additive non-decision time component, $t_0=0.1, 0.5, 1, 2$. Relative to the mean decision time (i.e. the mean response time without the additive non-decision component) of 1.1s, an additive non-decision component of 2s represents an extreme case where non-intrusive non-decision processes triple the observable decision time. All fitting procedures were the same as in the first simulation.

**Fig. 3:**
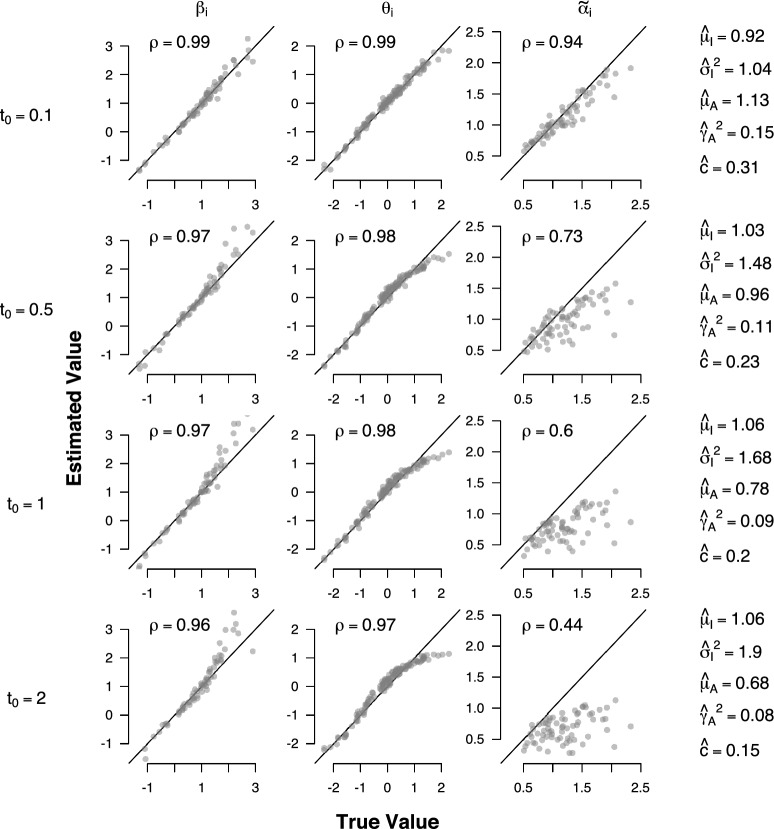
Parameter recovery simulation under additive non-decision time. Plots show the relationship between true and estimated values of the ABDM parameters when the generating model includes an additive non-decision time component. The generating population-level parameters were $\mu _I=1$, $\sigma ^2_I=1$, $\mu _{A}=1$, $\gamma ^2_{A}={1}/{2}$, and $c=0.35$.

Figure 3 shows how the additive non-decision component affects estimates of the ABDM parameters. Correlations $\rho$ between generating and estimated parameter values are given in the top left corner of each plot. As can be seen, item effects on the rate of information processing $\beta _i$ (leftmost column) are largely unaffected by $t_0$; but only extreme values of $t_0$ lead to a noticeable overestimation of large values of $\beta _i$. This is also confirmed by the overall high correlation between generating and estimated parameter values. Person effects on the rate of information processing $\theta _p$ (second column from the left) are largely unaffected by small values of $t_0$. Large values of $t_0$ result in an underestimation of large values of $\theta _p$ and an overestimation of small values of $\theta _p$. However, the bias is monotonic, which means that the order of $\theta _p$ across persons is not affected. Hence, even in the presence of an unrealistically large additive non-decision component, inferences about the relative rate of information processing among test takers are still valid. This is further confirmed by the overall high correlation between generating and estimated parameter values. Item effects on the volatility parameter $\tilde{\alpha }_i$ (third column from the left) are largely unaffected by very small values of $t_0$. However, for larger values of $t_0$, the $\tilde{\alpha }_i$ tend to be underestimated and the precision with which the generating value can be recovered decreases. This is also reflected in a sizeable decrease in the correlation between generating and estimated parameter values for large values of $t_0$. Finally, estimates of the population-level parameters (rightmost column) are largely unaffected by small values of $t_0$. For extreme values of $t_0$, $\mu _I$ remains largely unaffected, whilst the variance $\sigma ^2_I$ is overestimated, and $\mu _A$ and $\gamma ^2_A$ are considerably underestimated. Estimates of the boundary-to-volatility ratio *c* are unaffected for small values of $t_0$ but tend to be underestimated for larger values of $t_0$.

Taken together, the results of these simulations show that neglecting realistic, small additive non-decision components in the ABDM does not significantly bias parameter estimates. Large additive non-decision components mainly affect estimates of item volatility. However, these biases only become significant when the additive non-decision component is at least as large as the mean decision time. Substantively, this means that non-intrusive non-decision processes such as stimulus encoding and response execution would need to occupy as much time as decision processes, which is implausible in realistic psychometric settings.

## Application

We gauged the performance of our ABDM in realistic settings by re-analysing data sets used in Van Rijn and Ali (2017) and Molenaar et al. (2015). Since Molenaar et al.’s (2015) data set was considerably smaller, it provides little insight into the ABDM’s performance in the large-data contexts for which the model was developed. Therefore, we only report our analysis of Van Rijn and Ali’s (2017) data here, the analysis of Molenaar et al.’s (2015) data can be found in Appendix C. The data set from Van Rijn and Ali (2017) consists of RT and accuracy data from 4899 first-year students in teachers college who completed 50 spelling items as part of a Dutch language test. Items had a two-alternative forced choice format and were presented in random order. There were no time limits imposed for the completion of individual items, but the duration of the entire language test was limited to 90 min. For our analysis, we only used data from the A version of the test and excluded test takers with incomplete data, who spent less than 3 s per item on average, or had taken (a version of) the test previously.

**Fig. 4:**
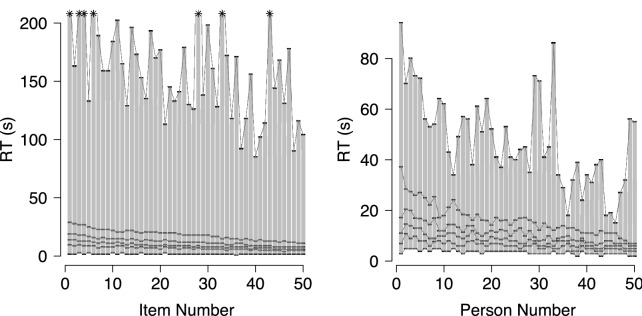
Marginal RT quantiles (0, 0.2, 0.4, 0.6, 0.8, and 1.0) for items (left plot) and 50 persons (right plot). Data are ordered by mean RT. Maximum RTs exceeding 200 s are indicated by asterisks.

Figure 4 shows the marginal RT quantiles (0, 0.2, 0.4, 0.6, 0.8, and 1.0) for each of the 50 items and for the first 50 persons, ordered by mean RT. As can be seen, marginal RT distributions for the items (left plot) had a sharp leading edge and a typical long tail. Minimum RTs were generally small compared to the median RT; the ratio of the minimum observed RT to the median RT ranged between 0.11 and 0.29, with a mean of 0.21. Since item-specific additive non-decision components cannot exceed the minimum RT, this means that, in line with the assumptions of our ABDM, any possible additive non-decision components were negligible relative to the median RT. Marginal RT distributions for the persons (right plot) also exhibited the typical skewed shape with a long tail. The leading edge tended to be less steep compared to the marginal distributions for the items. However, due to the small number of items, the exact shape of the marginal distributions is insufficiently constrained by the data. Although the ratio of the minimum observed RT to the median RT across all 4899 participants ranged between 0.10 and 0.80, for 90% of the participants the minimum RT was smaller than half their median RT. This means that any potential person-specific additive non-decision components were small relative to the median RT.

We aimed to compare the performance of our ABDM to Van der Linden’s (2009) popular hierarchical model and to Van der Maas et al.’s (2011) Q-diffusion model. We compared our ABDM to the two competitor models in terms of relative and absolute model fit. For the comparison of the relative model fit with the hierarchical model, we fitted both models to the full data set of 4899 persons and 50 items. For the comparison of the absolute model fit, we only used the data of the first 50 persons on all 50 items due to the formidable computational costs of generating data from the full ABDM. For the comparison of the relative as well as the absolute model fit with the Q-diffusion model, we only used the data of the first 200 persons and the first 15 items due to prohibitively long computing times of the Q-diffusion model (see also Van Rijn & Ali, 2017). Moreover, as the DM likelihood function approaches zero quickly for RTs above 10s, we fitted all models to the RTs in tenths of seconds to guarantee numerical stability.

Van der Linden’s (2009) hierarchical model describes log-RTs as normally distributed, where the RT for a person completing one item is described by a person-dependent speed parameter, and item-dependent discrimination and time-intensity parameters. The probability of giving a correct response is described by a normal ogive model with a person-dependent ability parameter and two item-dependent discrimination and difficulty parameters. Dependencies between RTs and accuracies are introduced through multivariate normal population-level distributions for the item and person parameters. Hence, the model as implemented by Fox et al. (2019) describes a data set with $N_I$ items and $N_P$ persons in terms of $2N_I+5N_P+25$ parameters.

Van der Maas et al.’s (2011) Q-diffusion model describes the joint distribution of RTs and accuracies in terms of the classic DM. The Q-diffusion model describes the rate of information processing and boundary separation as a ratio of a person and an item parameter. Moreover, non-decision time is assumed to be an additive component of the observed RT and is determined by an item parameter. Together with two parameters for the population-level variance of the person parameters, the model describes a data set with $N_I$ items and $N_P$ persons in terms of $3N_I+2N_P+2$ parameters.

For the comparison with the hierarchical model, we fitted our ABDM using the routine described earlier. We first estimated the boundary-to-volatility ratio *c* to using the golden section algorithm, where the algorithm terminated if either a maximum number of ten iterations had been reached or the change in the log-likelihood between iterations was less than 1%. To estimate the person and item parameters for each candidate value of *c*, we drew 2,000 posterior samples for all other model parameters and discarding the first 1,000 samples as burn-in samples. The golden section estimation of *c* was completed after seven iterations with a change in the log-likelihood of less than 0.03%. In a second step, we obtained the desired number of posterior samples for the best-fitting value of *c*. We sampled three chains with 12,000 posterior samples each of which we discarded the first 2,000 samples as burn-in. All chains showed good convergence, with $\hat{R} < 1.02$. We used the ddiffusion function implemented in the rtdists R package (Singmann et al., 2016) to compute the ABDM likelihood. We fitted van der Linden’s hierarchical model using the LNIRT function in Fox et al.’s (2019) LNIRT R package. We obtained three chains with 12,000 posterior samples each of which we discarded the first 2,000 samples as burn-in. All chains showed good convergence, with $\hat{R} < 1.02$.

We assessed relative model fit by means of AIC, BIC and DIC. For the computation of AIC and BIC, we evaluated the conditional model likelihoods^1^ at the posterior means. We also based our computation of DIC on the conditional likelihood for both models.^2^ Due to the long computing times required for the evaluation of the full ABDM likelihood function, we based the computation of the model selection criteria on the first 1,000 posterior samples after burn-in from a single MCMC chain. Table 2 shows the relative fit of the two models. As can be seen, the log-likelihood was larger for the ABDM than for the hierarchical model and all three model selection criteria indicated that the ABDM also fitted the data better when model complexity was taken into account.

**Table 2:** Relative model fits.

	AIC	BIC	DIC	Log-likelihood	No. of parameters
ABDM	695,714.2	747,799.8	690,607.5	$-$342,853.1	5,004
Hierarchical model	906,733.2	1,011,581.0	936,987.5	$-$443,293.6	10,073

We assessed the absolute model fit by means of the posterior predictive mean RTs and accuracies for persons and items. Due to the high computational costs of generating samples from the full ABDM, we limited our analysis to the first 50 items and persons in the data set. We generated 500 posterior predictive samples for each item and person. We used the rdiffusion function in the rtdists R package (Singmann et al., 2016) to generate posterior predictive samples for the ABDM, and we used custom-written code to generate posterior predictive samples for the hierarchical model.

**Fig. 5:**
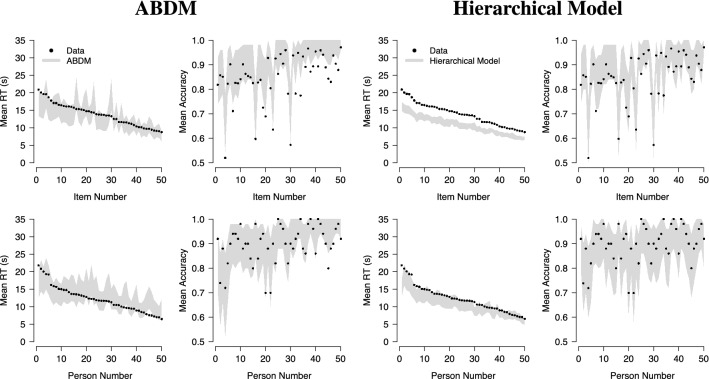
Posterior predicted mean RTs and accuracies for 50 items and persons. Results are ordered by mean RT. Results for the ABDM are shown in the left panel, results for the hierarchical model are shown in the right panel. Intervals indicate the range between the 0.025 and the 0.975 quantile of the posterior predictive values.

Figure 5 shows the comparison of the data and the posterior predictive samples generated by the two models, ordered by the mean RT in the data. As can be seen, the ABDM generated wider posterior predictive intervals for RTs (left column in the left panel) than the hierarchical model (left column in the right panel). Whereas the ABDM’s posterior predictives matched the mean item RTs relatively closely (top left plot in the left panel), the hierarchical model systematically underpredicted the mean item RTs (top left plot in the right panel). The ABDM overpredicted some of the mean person RTs (bottom left plot in the left panel), whereas the hierarchical model matched the mean person RTs closely (bottom left plot in the right panel). Both models matched the mean item (top right plot in each panel) and person accuracies (bottom right plot in each panel) closely, although the ABDM overpredicted some particularly low item accuracies. Taken together, both models fitted the data well, although the hierarchical model showed a systematic overprediction of mean item RTs that was not present in the ABDM.

As there are no Bayesian implementations available of the Q-diffusion, we developed a maximum-likelihood fitting method for our ABDM to compare both models on an equal footing. To avoid the computationally costly evaluation of the full ABDM likelihood required for the maximum-likelihood estimation of the boundary-to-volatility ratio *c*, we fixed *c* to the value estimated based on the complete data set in our comparison with the hierarchical model.

Joint maximum-likelihood estimates of the ABDM parameters can be obtained by differentiating the logarithm of the likelihood (6) with respect to the person and item effects on the rate of information processing and volatility, which yields:

$$\begin{aligned} \frac{\partial }{\partial \beta _i} \log \left( f(\vec {t},\vec {x} \mid \Theta ) \right)&= \sum _{p=1}^{N_P} \frac{x_{ip}-1/2}{c}\tilde{\alpha }_i - t_{ip}(\beta _i + \theta _p)\tilde{\alpha }^2_i \\ \frac{\partial }{\partial \theta _p} \log \left( f(\vec {t},\vec {x} \mid \Theta ) \right)&= \sum _{i=1}^{N_I} \frac{x_{ip}-1/2}{c}\tilde{\alpha }_i - t_{ip}(\beta _i + \theta _p)\tilde{\alpha }^2_i \\ \frac{\partial }{\partial \tilde{\alpha }_i} \log \left( f(\vec {t},\vec {x} \mid \Theta ) \right)&= \sum _{p=1}^{N_P} \frac{x_{ip}-1/2}{c}(\beta _i + \theta _p) - t_{ip}(\beta _i + \theta _p)^2\tilde{\alpha }_i. \end{aligned}$$

Equating to 0 and solving for $\beta _i$, $\theta _p$, and $\tilde{\alpha }_i$ yields for the estimators:

$$\begin{aligned} \hat{\beta }_i&= \frac{\sum _{p=1}^{N_P} (x_{ip}-1/2) - ct_{ip}\theta _p \tilde{\alpha }_i}{c\sum _{p=1}^{N_P} t_{ip} \tilde{\alpha }_i} \\ \hat{\theta }_p&= \frac{\sum _{i=1}^{N_I} (x_{ip}-1/2) - ct_{ip}\beta _i \tilde{\alpha }_i}{c\sum _{p=1}^{N_P} t_{ip} \tilde{\alpha }_i} \\ \hat{\tilde{\alpha }}_i&= \frac{\sum _{p=1}^{N_P} (x_{ip}-1/2)}{c\sum _{p=1}^{N_P} t_{ip} (\beta _i + \theta _p)}, \end{aligned}$$

which can be solved by iterative computation. To identify the model, we imposed the same constraints on the $\theta _p$ as we imposed on the population mean and variance in our Bayesian implementation, fixing the mean and variance of the $\theta _p$ to $\bar{\theta } = {1}/{N_P}\sum _{p=1}^{N_P}\theta _p = 0$ and $\sigma ^2 = {1}/{N_P}\sum _{p=1}^{N_P}(\theta _p-\bar{\theta })^2 = 1$. Note that the remaining population-level parameters, $\mu _I, \sigma ^2_I, \mu _A$, and $\gamma ^2_A$, are not estimated in the JML approach. We obtained maximum-likelihood estimates of the Q-diffusion parameters using the diffIRT function in the diffIRT R package (Molenaar, 2015). We fitted both models to RTs divided by 10 to guarantee numerical stability when evaluating the model likelihood. One reviewer pointed out that the selection of the 15 items might have affected the outcomes of the model comparisons. We therefore repeated the model comparisons with a different set of 15 items. As the results of both analyses agreed closely, we only report the results for the first set of 15 items. The analysis for the second set of 15 items is reported in Appendix D.

We assessed relative model fit by means of AIC and BIC. Table 3 shows the relative fit of the two models. As can be seen, the log-likelihood was smaller for the ABDM than for the Q-diffusion model. AIC preferred the Q-diffusion model over the ABDM, whereas BIC preferred the ABDM over the Q-diffusion model. This difference between the two criteria is due to the higher penalty for model complexity imposed by BIC, which thus avoids selecting overly complex models at small sample sizes. Moreover, the difference in AIC between the two models was considerably smaller than the difference in BIC. These results suggest that the ABDM accounted as well for the data as the Q-diffusion model when model complexity was taken into account.

**Table 3:** Relative model fits.

	AIC	BIC	Log-likelihood	No. of parameters
ABDM	7,994.8	9,370.2	$-$3,768.4	229
Q-Diffusion	7,679.7	10,364.6	$-$3,393.0	447

We assessed absolute model fit through a cross-validation approach. To this end, we split up the data in two ways. Firstly, we divided the data into five folds of 40 persons each, removed one fold from the data and fitted both models to the remaining data (person-based fit). Secondly, we divided the data into five folds of three items each, removed one fold from the data and fitted both models to the remaining data (item-based fit). We used the results of the person-based fit to compute the item parameters for the item fold removed in the item-based fit, and we used the results of the item-based fit to compute the person parameters for the person-fold removed in the person-based fit. We subsequently combined the person and item parameters obtained in the two steps and used expressions (2) and (3) to predict RTs and accuracies for the person-by-item combinations contained in the removed folds. This was repeated for all 25 person-by-item folds.

**Fig. 6:**
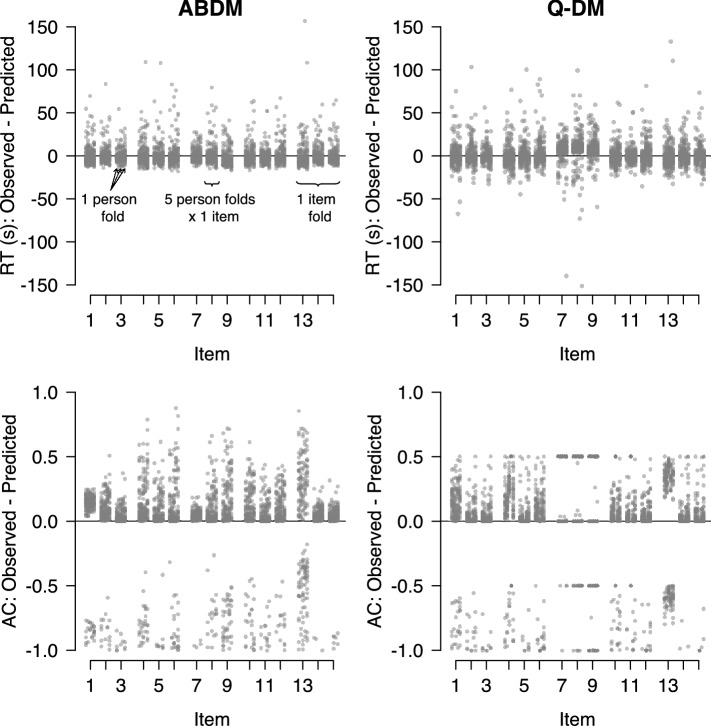
Out-of-sample predictions in fivefold cross-validation for the ABDM and the Q-diffusion model. Predictions are based on data from 200 persons and 15 items. Results for the ABDM are shown on the left results for the Q-diffusion model are shown on the right. Each column of dots shows the results for one person fold, each cluster of five columns shows the results for all five person folds in one item, and each group of three items shows the results for all five person folds on one item fold.

Figure 6 shows the deviation between observed and predicted RTs (top row of plots) and accuracies (bottom row of plots) for the ABDM (left column of plots) and the Q-diffusion model (right column of plots). Each column of dots shows the results for the 40 persons in one person-fold, each cluster of five columns of dots shows the results for all five person folds on one item, and each group of three items shows the results for all five person folds in one item fold. As can be seen, the prediction errors for RTs for the ABDM clustered symmetrically around 0, with a few large positive outliers. This means that the ABDM predicted RTs were largely unbiased, except for very long RTs, in which case the ABDM tended to underpredict observed RTs. For the Q-diffusion model, prediction errors also clustered symmetrically around 0, except for the third item fold, for which the model systematically underpredicted the observed RTs. The spread of the prediction error for the Q-diffusion model was comparable to that for the ABDM. Finally, the Q-diffusion model showed a less pronounced tendency to underpredict long RTs than the ABDM; the Q-diffusion model equally often overpredicted short RTs.

The results for the predicted accuracy are again similar between the two models. For the ABDM prediction, errors tend to be positive, which indicates a tendency to underpredict accuracies. Nevertheless, most prediction errors are close to 0, which means that the ABDM generally captured the probability of a correct response well. The Q-diffusion model showed a similar tendency to underpredict accuracies but, in general, predicted the probability of a correct response well, with most prediction errors being slightly larger than 0. The spread of the prediction errors is also comparable to those observed for the ABDM. However, for the third item fold, the Q-diffusion model showed a systematic underprediction of accuracies. Taken together, both models provided a comparable absolute fit to the data.

To sum up, our comparison with the hierarchical model showed that our ABDM provides a better relative fit and a comparable absolute fit to the data, with a considerably smaller number of parameters. Our comparison with the Q-diffusion model further showed that the ABDM provides a comparable relative and absolute fit to the data. This latter result indicates that our ABDM does not lose much descriptive power compared to an instantiation of the classic DM, but requires fewer parameters ($N_I+N_P+4$, to be precise).

### Regression Model

As a final step in the assessment of our ABDM, we tested whether the estimated person effects on drift rate behave in accordance with their substantive interpretation. Van Rijn and Ali’s (2017) data set included information on test takers’ prior education. If individual differences in the person effects on drift rate $\theta _p$ do indeed reflect differences in the speed of information processing, one would expect that $\theta _p$ is larger for test takers who completed a higher level of prior education. To test this substantive hypothesis, we extended our ABDM with a regression model that described the $\theta _p$ in terms of the dummy-coded prior education levels, with MBO as the reference level.

**Fig. 7:**
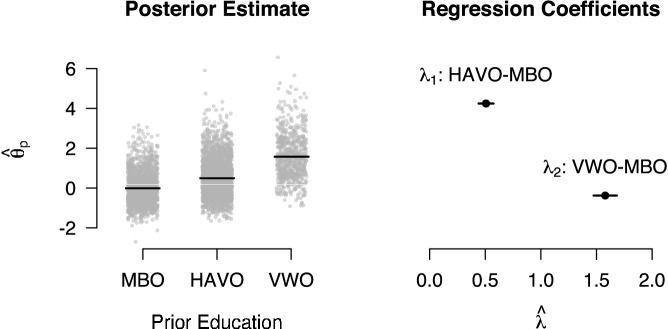
Relationship between person effect on drift rate and prior education. Posterior means from the model without regression component are shown on the left. Data are jittered for improved visibility. Horizontal lines indicate the group means. Posterior means (dots) and 95% equal-tailed credible interval (lines) for the regression coefficients are shown on the right.

Prior education was categorised as MBO, HAVO, or VWO, which reflect increasing levels in the Dutch education system. We excluded the data of 348 test takers from this analysis as there were either no data available on their prior education or their prior education did not fall into one of the three categories. As a first analysis step, we fitted the ABDM without the regression component to the data. The left panel in Fig. 7 shows the posterior means of $\theta _p$ for the three education levels. As can be seen, the estimated person effects were smallest for test takers with MBO as their prior education and largest for test takers with a VWO education. As a second step, we fitted the ABDM with the regression extension to the data. The right panel in Fig. 7 shows the equal-tailed 95% credible intervals for the regression coefficients. In line with the hypothesis, the regression coefficient for the difference between HAVO and MBO was positive and the credible interval did not include 0. The difference between VWO and MBO was considerably larger than the difference between HAVO and MBO, and the credible interval again did not include 0. Taken together, these results are in line with the substantive hypothesis that higher education levels are associated with faster information processes, which is represented by the person effects on drift rate.

## Discussion

In the present paper, we developed the Attention-Based Diffusion Model. Based on cognitive process assumptions that we believe to be appropriate in psychometric contexts, we derived a substantively meaningful model with favourable computational properties. Our simulation study demonstrated good parameter recovery and furthermore showed the robustness of our model to the presence of non-intrusive non-decision processes. In our application to data from a performance test, we compared our ABDM to Van der Linden’s (2009) popular hierarchical model and to Van der Maas et al.’s (2011) Q-diffusion model. Whereas our ABDM and the hierarchical model could be fitted to the complete data set, we had to limit our comparison with the Q-diffusion model to a subset of the data due to the considerable computing time required to fit the Q-diffusion model to data. Formal model comparisons showed that our ABDM provided a better fit to the data than the hierarchical model and performed as well as the Q-diffusion model. These results indicate that our ABDM provides a substantively meaningful and computationally efficient model for performance test data.

One limitation on the theoretical interpretation of our ABDM is the requirement that data conform to a two-alternative forced choice format, where each decision threshold corresponds to one response option and the sign of the rate parameter indicates which response option is favoured by the accumulated information. However, this is rarely the case in psychometric settings, where items typically have a multiple-choice format. In a multiple-choice setting, there is no such clear correspondence between the response options, and the two decision thresholds and the sign of the drift rate parameter. In some testing situations, this problem might be resolved by grouping response options. Specifically, if there is only one correct option and the incorrect options are equally attractive, that is, each incorrect option generates information at the same rate, all incorrect options might be subsumed under one threshold and the correct response might be associated with the other threshold (see also Van der Maas et al., 2011). However, in a setting with multiple correct answers that differ in their attractiveness, as is typically the case in attitude or personality tests, models based on a diffusion process with two thresholds will generally not be applicable (e.g. Ratcliff et al., 2016).

An interesting theoretical aspect of our ABDM is the fixed volatility-to-response caution ratio. This assumption corresponds to linearly decreasing decision boundaries (Hanks et al., 2011). In the classic DM, the assumption that decision boundaries are constant is motivated by a statistical optimality argument; constant boundaries yield the lowest mean decision time for a given accuracy level if the stimulus strength and volatility are constant (Wald & Wolfowitz, 1948). However, in natural environments, as well as in psychometric settings, stimulus strength and/or volatility varies between decisions. Under these circumstances, decision makers should become increasingly willing to sacrifice decision accuracy as the decision time increases to avoid unacceptably long mean decision times. An optimal trade-off between decision time and accuracy can be achieved by decreasing the decision boundaries (Anderson, 1960; Shadlen & Kiani, 2013).

An important criterion in the development of latent trait models for RT data is the trade-off between statistical convenience and substantive scope. At one end of the spectrum are highly tractable statistical models that are void of any conceptual commitments. At the other of the spectrum are models from mathematical psychology that are conceptually rich but computationally inefficient. Purely statistical models are unsatisfactory as psychological theories. Because the validity of psychometric tests hinges on the psychological processes that connect item presentation and item response, models should provide a substantively meaningful account of this connection (Van der Maas et al., 2011). Although models from mathematical psychology provide this type of substantively meaningful item response theory, their functional form precludes applications of models from mathematical psychology to large psychometric data sets. To attain an acceptable trade-off between computational efficiency and substantive scope, researchers typically remove conceptual commitments that are embodied in the model’s functional form until the resulting model is sufficiently tractable for psychometric applications. In recent years, several authors have suggested such simplified cognitive models for psychometric applications. In the remainder of this section, we will discuss how the trade-off between statistical convenience and substantive scope in our ABDM compares to the trade-offs made in these models.

Our development of the ABDM here is based on a diffusion process account of item responding with substantive assumptions that are adjusted to a psychometric context. A close relative of our ABDM is the proportional-rate diffusion model suggested by Palmer et al. (2005). They assume unbiased responding and a constant ratio of response caution to volatility which, unlike our ABDM, they fix to 1. Moreover, Palmer et al. assume that drift rate is a constant multiple of the known stimulus strength in a random dot motion task. These simplifying assumptions result in a two-parameter model for psychophysical applications.

Other researchers have attempted to make the classic DM applicable to psychometric data whilst retaining the model’s substantive commitments. Van der Maas et al.’s (2011) Q-diffusion model imposes a random effects structure on the four parameters of the classic DM for psychometric applications. A more flexible version of the classic DM is provided by Vandekerckhove et al’s. (2010) hierarchical Bayesian diffusion model (HDM), which allows users to implement custom random effects and regression components. Although both models provide a statistical structure that is appropriate for psychometric applications, their substantive assumptions seem to be inappropriate in psychometric contexts. Moreover, the functional form of the classic DM used in HDM and Q-diffusion is computational intractable and thus precludes applications in psychometric settings with large data sets, as demonstrated in our example application.

A further model that is based on the classic DM is Wagenmakers et al. (2007) EZ model. The EZ model uses moment estimators to efficiently fit a three-parameter version of the classic DM, under the assumption that the starting point of the accumulation process is equidistant from the decision thresholds. Estimation of the model parameters is highly efficient as it relies on closed-form expressions for the model parameters in terms of the probability of a correct response and the mean and variance of correct RTs. However, in addition to the questionable appropriateness of the model’s process assumptions in psychometric contexts, the reliance on the moments of the marginal likelihood functions for RT and accuracy severely limits the model’s flexibility. The three model parameters can only be estimated from either the marginal RT distribution for each item or the marginal RT distribution for each test taker, which precludes the estimation of separate person and item effects (see also Ratcliff, 2008). Moreover, the lack of a likelihood function means that the model cannot be implemented hierarchically or extended in any meaningful way.

Another class of cognitive models that have been adapted for psychometric applications are ballistic accumulator models. These models replace the stochastic information accumulation process in the DM by a ballistic process and allow information to accumulate independently for each choice option, which yields more tractable models (e.g. Brown & Heathcote, 2008). One particular instantiation of a ballistic accumulator model, the log-normal race model (LNR; Heathcote & Love, 2012), has recently been adapted to account for RTs in attitudinal and personality tests with multiple response categories (Ranger & Kuhn, 2018). Moreover, Rouder et al.’s (2015) hierarchical Bayesian implementation of the LNR can account for RT data in ability tests with multiple response options. Their model can furthermore be extended to include autocorrelation and latent regression components and is straightforward to analyse using Gibbs sampling. However, although the LNR model parameters have a cognitive process interpretation similar to the DM parameters, the LNR model lacks the key distinction between boundary separation and the rate of information accumulation. Thus, whilst the LNR model shares the statistical convenience of our ABDM and can readily be applied in settings with more than two choice options, the lack of a differentiation between boundary separation and the rate of information accumulation represents a considerable limitation of the substantive scope of the LNR.

Taken together, our ABDM combines a high level of computational efficiency and statistical flexibility with a conceptually meaningful interpretation of its model parameters. It thus outperforms earlier attempts to make diffusion-type models applicable in psychometric contexts. Within the larger class of accumulator models of decision-making, only the LNR model provides a similar level of statistical convenience but has a more limited substantive scope.

## Figures and Tables

**Fig. 8:**
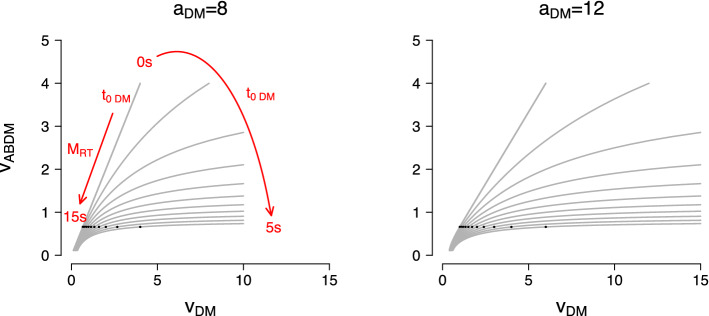
Relationship between parameters of the DM and the ABDM. Solid lines show how the drift rate in the ABDM, $v_{ABDM}$, relates to the drift rate in the DM, $v_{DM}$. Each grey line shows the relationship between the drift rate parameters of the two models for a fixed value of non-decision time $t_{0_{DM}}$ in the classic DM. The left panel shows the results for a boundary separation value of $a_{DM}=8$ in the classic DM; the right panel shows the results for $a_{DM}=12$. Small black dots show the drift rate values in each model that correspond to a mean response time of 6 s. Drift rates for the ABDM are shown for a diffusion coefficient of $s_{ABDM}=1$ and ratio of boundary separation to drift rate of $c=1/8$. The bias parameter in the classic DM was set to $z_{DM}=a_{DM}/2$.

**Fig. 9:**
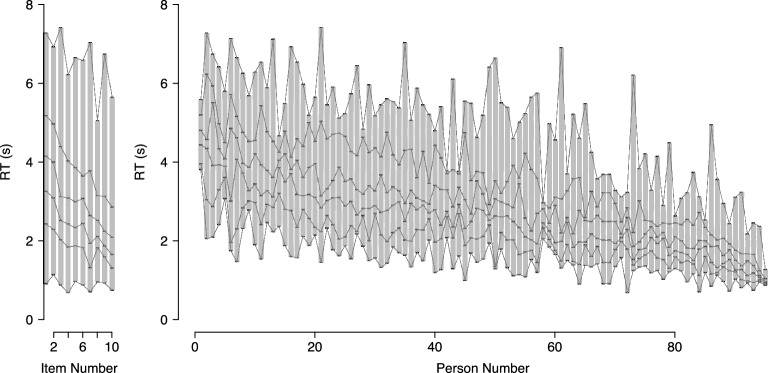
Marginal RT quantiles (0, 0.2, 0.4, 0.6, 0.8, and 1.0) for items (left plot) and 50 persons (right plot). Data are ordered by mean RT.

**Fig. 10:**
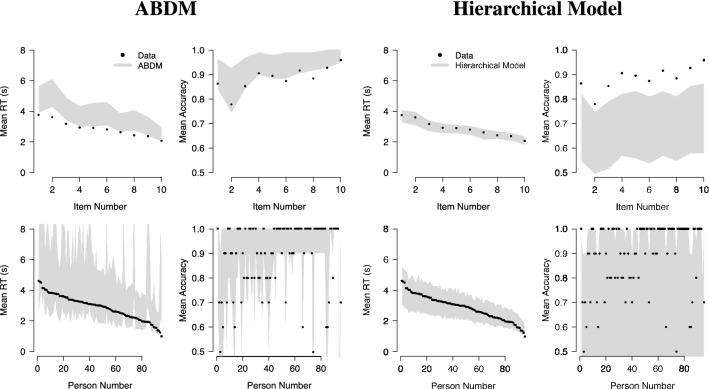
Posterior predicted mean RTs and accuracies. Results are ordered by mean RT. Results for the ABDM are shown in the left panel, and results for the hierarchical model are shown in the right panel. Intervals indicate the range between the 0.025 and the 0.975 quantile of the posterior predictive values.

**Fig. 11:**
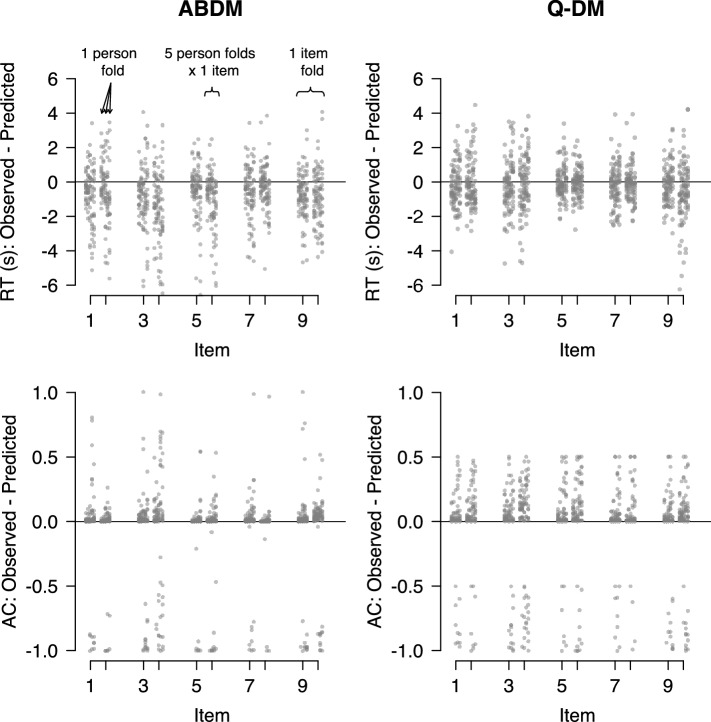
Out-of-sample predictions in fivefold cross-validation for the ABDM and the Q-diffusion model. Predictions are based on data from 200 persons and 15 items. Results for the ABDM are shown on the left panel, and results for the Q-diffusion model are shown on the right. Each column of dots shows the results for one person fold; each cluster of five columns shows the results for all five person folds on one item, and each group of three items shows the results for all five person folds on one item fold.

**Fig. 12:**
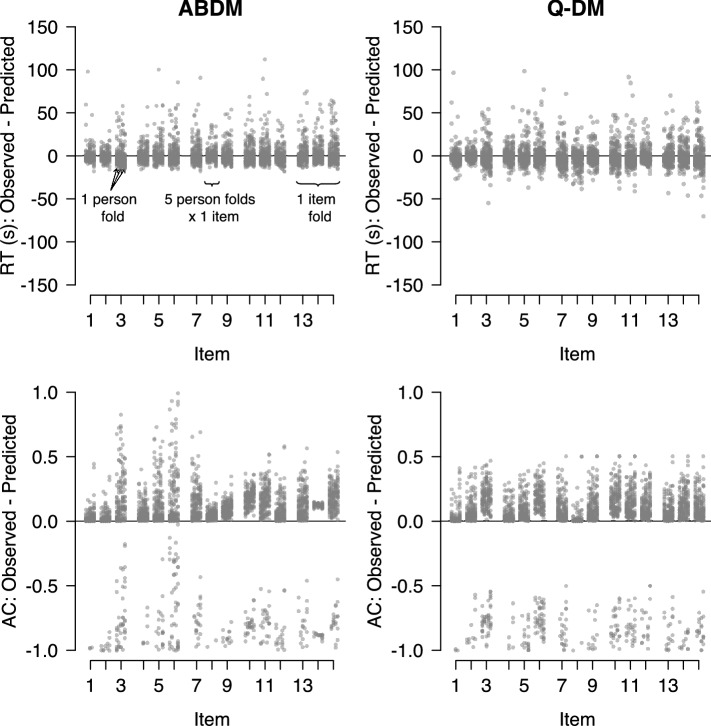
Out-of-sample predictions in fivefold cross-validation for the ABDM and the Q-diffusion model. Predictions are based on data from 200 persons and a different set of 15 items from the main text. Results for the ABDM are shown on the left panel, and results for the Q-diffusion model are shown on the right. Each column of dots shows the results for one person fold, and each cluster of five columns shows the results for all five person folds on one item, and each group of three items shows the results for all five person folds on one item fold.

## Appendix A: Non-Decision Time in the ABDM

In our ABDM, we assume that lapses in attention affect decision processes, decreasing the effective rate of information accumulation. Our conceptualisation of these attentional lapses can be most easily understood by contrasting the random walk approximation of the classic DM with the random walk approximation of our ABDM. We begin by showing that the diffusion process in the classic DM is the weak limit of a random walk in which the walker moves either up or down at each time step. We base our exposition on Schilling and Partzsch (2014).

We discretise space into units of size $\Delta x$ and consider discrete time steps of size $\Delta t$ in an interval [0, *T*]. This means that there are $N = \lfloor {T}/{\Delta t}\rfloor$ time steps in total, where $\lfloor \cdot \rfloor$ denotes rounding down to the nearest integer. Let $\mathscr {E}_n \sim \text {Bern}(p)$, $n = 1, \ldots , N$ be independent Bernoulli random variables with parameter $p \in [0,1]$ and define $X_n = 2\mathscr {E}_n-1$. At each time step *n*, the walker moves $X_n \Delta x$ units up or down, depending on the sign of $X_n$. The random walk process is defined as:

A1 $$\begin{aligned} X(T) = \sum _{n=1}^N X_n \Delta x. \end{aligned}$$

The mean and variance of *X*(*T*) are:

A2 $$\begin{aligned} \mathbb {E}[X(T)]&= N\Delta x (2p-1)\nonumber \\ \mathbb {V}[X(T)]&= N(\Delta x)^2 4p(1-p). \end{aligned}$$

We now note that (i) $X(0) = 0$. If we let $T' = M\Delta t$, $M < N$, we may write $X(T) = (X(T)-X(T')) + (X(T')-X(0))$. Since the $\mathscr {E}_n$ are independent, identically distributed random variables, the increments $X(T)-X(T')$ and $X(T')-X(0)$ are also (ii) independent and (iii) identically distributed random variables. These are three of the four properties we require for *X*(*T*) to approximate a diffusion process as $N\rightarrow \infty$. The last property we require is that increments must be normally distributed with variance $(T-T')\sigma ^2$, where the constant $\sigma$ is the diffusion coefficient. To show this, we first note that independence of the increments implies that $\mathbb {V}[X(T)]=\mathbb {V}[X(T)-X(T')]+\mathbb {V}[X(T')-X(0)]$, that is, the variance of *X*(*T*) is linear in *T*:

$$\begin{aligned} \mathbb {V}[X(T)] = T\sigma ^2. \end{aligned}$$

From Eq. (A2), we conclude that:

$$\begin{aligned} \sigma ^2 = \frac{(\Delta x)^2}{\Delta t}4p(1-p). \end{aligned}$$

We now write:

A3 $$\begin{aligned} X(T) = \frac{X(T) - N\Delta x (2p-1)}{\sqrt{T\sigma ^2}}\sqrt{T\sigma ^2} + N\Delta x (2p-1), \end{aligned}$$

and let $p = {1}/{2} \left( \mu \left( {\Delta t}/{\Delta x}\right) +1 \right)$, where the constant $\mu$ is the drift rate. Taking $N\rightarrow \infty$ and applying the central limit theorem to the first term shows that (iv) the limiting process has normally distributed increments with mean $T \mu$ and variance $T\sigma ^2$. This shows that the process *X*(*T*) approximates a diffusion process.

In contrast to the classic DM, the random walk approximation to our ABDM allows the random walker to remain at its current position with a certain probability at each time step. Nevertheless, the weak limit of this random walk is again a diffusion process.

Using the same discretisation of space and time as above, we now consider two sets of independent Bernoulli random variables $\mathscr {E}_{+,n} \sim \text {Bern}(p_+)$ and $\mathscr {E}_{-,n} \sim \text {Bern}(p_-)$ with $n = 1, \ldots , N$ and $p_{\pm } \in [0,1]$. We now define $Y_n = \Delta x (\mathscr {E}_{+,n} - \mathscr {E}_{-,n})$ and note that:

$$\begin{aligned} \mathbb {P}(Y_n = \Delta x)&= p_+(1-p_-) \\ \mathbb {P}(Y_n = 0)&= p_+p_-+(1-p_+)(1-p_-) \\ \mathbb {P}(Y_n = -\Delta x)&= p_-(1-p_+). \end{aligned}$$

That is, at each time step *n* the walker moves $Y_n \Delta x$ units up, down, or remains at its current position, depending on the value of $Y_n$. The corresponding random walk process is defined as:

A4 $$\begin{aligned} Y(T) = \sum _{n=1}^N Y_n \Delta x. \end{aligned}$$

The mean and variance of *Y*(*T*) are:

A5 $$\begin{aligned} \mathbb {E}[Y(T)]&= N\Delta x (p_{+}-p_{-})\nonumber \\ \mathbb {V}[Y(T)]&= N(\Delta x)^2 \left( p_{+}+p_{-}-(p_{+}^2+p_{-}^2)\right) . \end{aligned}$$

Clearly, (i) $Y(0) = 0$ and the independence of $\mathscr {E}_{+,n}$ and $\mathscr {E}_{-,n}$ means that *Y*(*T*) has (ii) independent and (iii) identically distributed increments. A similar argument as above furthermore shows that the variance of *Y*(*T*) is linear in *T* and:

$$\begin{aligned} \sigma ^2 = \frac{(\Delta x)^2}{\Delta t} \left( p_++p_--(p_+^2+p_-^2)\right) . \end{aligned}$$

We now write:

A6 $$\begin{aligned} Y(T) = \frac{Y(T) - N\Delta x (p_+-p_-)}{\sqrt{T\sigma ^2}}\sqrt{T\sigma ^2} + N\Delta x (p_+-p_-), \end{aligned}$$

and let $p_{\pm } = {1}/{2}\left( (1-p_0)\pm \mu \left( {\Delta t}/{\Delta x}\right) \right)$, where the constant $\mu$ is the drift rate and $p_0=p_+p_-+(1-p_+)(1-p_-)$ is the probability that the random walker does not move at some time step. Taking $N\rightarrow \infty$ and applying the central limit theorem to the first term shows that (iv) the limiting process has normally distributed increments with mean $T\mu$ and variance $T\sigma ^2$. This shows that the process *Y*(*T*) approximates a diffusion process. Moreover, the expression for the expectation in Eq. (A5) shows that information is accumulated at a lower rate if non-decision time is larger (i.e. for larger $p_0$).

## Appendix B: Relationship Between the Attention-Based and Classic Diffusion Model

Here, we explore how the parameters of the classic DM are related to the parameters of our ABDM. From the expression for the mean RT and the probability of choosing option A given in Eqs. (2) and (3), we can derive the corresponding expressions in our ABDM for the mean RT, $M_{RT~ABDM}$:

B1 $$\begin{aligned} M_{RT~ABDM} = \mathbb {E}(t) = \frac{s_{ABDM}}{2v_{ABDM}c} \frac{1-\exp {\left( -\frac{v_{ABDM}}{cs_{ABDM}}\right) }}{1 + \exp {\left( -\frac{v_{ABDM}}{cs_{ABDM}}\right) }}, \end{aligned}$$

and for the probability of choosing option A, $P_{A~ABDM}$:

B2 $$\begin{aligned} P_{A~ABDM} = \mathbb {P}(x=1) = \frac{1}{1+\exp {\left( -\frac{v_{ABDM}}{cs_{ABDM}}\right) }}. \end{aligned}$$

These latter expressions provide a simple way to study the relationship between our ABDM and the classic DM. Given a fixed value for *c*, say $c=1/8$, the ABDM includes only two free parameters, which, for the purpose of this comparison, are denoted $v_{ABDM}$ and $s_{ABDM}$. As can be seen from Eqs. (B1) and (B2), in the absence of further constraints on the model parameters, $M_{RT~ABDM}$ and $P_{A~ABDM}$ are only determined up to a ratio of $v_{ABDM}$ and $s_{ABDM}$. For our comparison with the classic DM, we will, therefore, assume that $s_{ABDM}=1$. Similarly, for this comparison we denote the parameters of the DM as $v_{DM}$, $s_{DM}$, $z_{DM}$, $a_{DM}$, and $t_{0_{DM}}$. We adopt the usual assumption that the diffusion coefficient^3^$s_{DM}=1$, and we furthermore assume that the decision process is unbiased, that is, $z_{DM}=\frac{a_{DM}}{2}$ (this corresponds to the EZ-diffusion model; Wagenmakers et al., 2007). Thus, the DM has three free parameters, $v_{DM}$, $a_{DM}$, and $t_{0_{DM}}$. The resulting expression in the DM for the mean response, $M_{RT~DM}$, is:

B3 $$\begin{aligned} M_{RT~DM} = \mathbb {E}(t) = t_{0_{DM}}+\frac{a_{DM}}{2v_{DM}} \frac{1-\exp {\left( -v_{DM}a_{DM}\right) }}{1 + \exp {\left( -v_{DM}a_{DM}\right) }}, \end{aligned}$$

and the expression for the probability of choosing option A, $P_{A~DM}$, is:

B4 $$\begin{aligned} P_{A~DM} = \mathbb {P}(x=1) = \frac{1}{1+\exp {\left( -v_{DM}a_{DM}\right) }}. \end{aligned}$$

We can now study the relationship between the two models by setting $M_{RT}=M_{RT~ABDM}=M_{RT~DM}$ to a fixed value, and numerically solving Eq. (B1) for $v_{ABDM}$ and numerically solving Eq. (B3) for $v_{DM}$ whilst $a_{DM}$ and $t_{0_{DM}}$ are held constant at different values.

Figure 8 shows the relationship between the ABDM parameters (x-axis) and the DM parameters (y-axis). The grey curves show how $v_{ABDM}$ relates to $v_{DM}$ as $M_{RT}$ decreases from 15 s (bottom left on each curve) to $t_{0_{DM}}$ s (top right on each curve). Each grey curve shows the relationship between $v_{ABDM}$ and $v_{DM}$ for a different fixed value of $t_{0_{DM}}$. Black dots show the values of $v_{ABDM}$ and $v_{DM}$ that produce a mean response time of $M_{RT}=6$ s. The left panel shows the relationship when $a_{DM}=8$; the right panel shows the relationship when $a_{DM}=12$. As can be seen, the qualitative pattern is the same for both values of $a_{DM}$: $v_{ABDM}$ and $v_{DM}$ increase monotonically as $M_{RT}$ increases from left to right along any of the grey curves. Higher values of boundary separation in the DM merely scale up the x-axis, which means that larger values of $v_{DM}$ are required to match the $M_{RT}$ produced by the ABDM. For instance, if $a_{DM}=8$ and $t_{0_{DM}}=0$ s (leftmost straight line in the left panel), a mean response time of $M_{RT}=6$ s is produced by the same drift rate of $v_{DM}=v_{ABDM}=0.66$ in the DM and in the ABDM. If $a_{DM}=12$ and $t_{0_{DM}}=0$ s (leftmost straight line in the right panel), a mean response time of $M_{RT}=6$ s is produced by a drift rate of $v_{DM}=1.00$ in the DM but only requires a drift rate of $v_{ABDM}=0.66$ in the ABDM.

The effect of $t_{0_{DM}}$ for a fixed value of $a_{DM}$ is to modulate the slope and curvature of the grey curves. For larger values of $t_{0_{DM}}$, the slope of the grey curves becomes shallower and small mean response times are produced by much smaller values of $v_{ABDM}$ compared to $v_{DM}$. For instance, if $a_{DM}=8$ (left panel) and $t_{0_{DM}}=0$ s, a mean response time of $M_{RT}=6$ s is produced by $v_{ABDM}=v_{DM}=0.66$. As the non-decision time increases to $t_{0_{DM}}=5$ s, the DM requires $v_{DM}=4$ to produce a mean response time of $M_{RT}=6$ s, whilst the ABDM produces the same mean response time with $v_{DM}=0.66$.

Taken together, in the case of non-response time close to 0 s, the drift rate parameters in both models are approximately linearly related. Hence, for small non-decision times the drift rate parameter in the ABDM can be interpreted analogously to the drift rate parameter in the classic DM. For higher values of boundary separation in the classic DM, the drift rate in the classic DM corresponds to a lower ratio of boundary separation and diffusion coefficient *c* in the ABDM. For larger values of non-response time, the relation between the drift rate parameters in both models becomes increasingly nonlinear as the drift rate parameter in the ABDM accounts for the combined effects of non-decision and decision processes. However, as already observed in our simulation studies, the relationship between the drift rate parameters in both models remains monotonic, which means that comparisons based on the ordering of different drift rate values in the two models remain valid.

## Appendix C: Analysis of Data Set II From Molenaar (2015)

Data set II from Molenaar (2015) consists of RT and accuracy data of 121 test takers answering ten items of a mental rotation test. Each item had a response deadline of 7.5s (Borst et al., 2011). Twenty-six persons failed to complete all items, and we excluded these person’s data from our analysis.

Figure 9 shows the marginal RT quantiles (0, 0.2, 0.4, 0.6, 0.8, and 1.0) for each of the ten items and for each of the 95 persons, ordered by mean RT. As can be seen, marginal RT distributions for the items (left plot) had a relatively shallow leading edge and a typical long tail. Compared to Van Rijn and Ali’s (2017) data, the minimum RTs in this data set were longer relative to the median RT; the ratio of the minimum observed RT to the median RT ranged between 0.25 and 0.45, with a mean of 0.35. Hence, item-specific additive non-decision components might have a more pronounced effect the RT data.

Marginal RT distributions for the persons (right plot) again had a shallow leading edge and exhibited a typical long tail. However, due to the small number of items, the exact shape of the marginal distributions is insufficiently constrained by the data. The ratio of the minimum observed RT to the median RT ranged between 0.28 and 0.93, with a mean of 0.58. This again means that person-specific additive non-decision components might significantly affect the RT data.

We compared the relative and absolute fit of our ABDM to Van der Linden’s (2009) hierarchical model and Van der Maas et al.’s (2011) Q-diffusion model in the same way as in the main text. For the comparison with the hierarchical model, we again used the golden section algorithm to estimate the boundary-to-volatility ratio *c* with the same termination criteria as before. We subsequently obtained 12,000 posterior samples from 3 MCMC chains for both models and discarded the first 2,000 samples as burn-in. All chains showed good convergence, with $\hat{R} < 1.01$ for both models.

We assessed relative model fit by means of AIC, BIC and DIC. Due to the long computing times required for the evaluation of the full ABDM likelihood function, we based the computation of the model selection criteria on the first 1,000 posterior samples after burn-in from a single MCMC chain. Table 4 shows the relative fit of the two models. As can be seen, the log-likelihood was smaller for the ABDM. AIC and BIC indicated that the hierarchical model fitted the data better than the ABDM, whereas DIC preferred the ABDM over the hierarchical model.

**Table 4:** Relative model fits.

	AIC	BIC	DIC	Log-likelihood	No. of parameters
ABDM	4,182.2	4,760.1	4,013.2	$-$1,972.1	119
Hierarchical Model	2,687.5	3,974.5	5,775.4	$-$1,078.8	265

We assessed the absolute model fit by means of the posterior predictive mean RTs and accuracies for persons and items. We generated 500 posterior predictive samples for each item and person. Figure 10 shows the comparison of the data and the posterior predictive samples generated by the two models, ordered by the mean RT in the data. As can be seen, the ABDM generated wider posterior predictive intervals for RTs (left column in the left panel) than the hierarchical model (left column in the right panel). Whereas the ABDM systematically underpredicted the mean item RTs (top left plot in the left panel), the hierarchical model’s posterior predictives matched the mean item RTs relatively closely (top left plot in the right panel). Both models’ posterior predictives covered the mean person RTs well (bottom left plot in the both panels). The results for the mean accuracies showed the opposite pattern. Whereas the ABDM matched the item and person accuracies well (right column in the left panel), the hierarchical model underpredicted both the mean item and person accuracies (right column in the right panel). Taken together, the hierarchical model provided a better fit to the RT data, whereas the ABDM provided a better fit to the accuracy data.

We compared our ABDM to the Q-diffusion model based on maximum-likelihood fits. We again used the estimate of the boundary-to-volatility ratio *c* from the Bayesian implementation of the ABDM to avoid the formidable computational costs associated with evaluating the complete ABDM likelihood.

We assessed relative model fit by means of AIC and BIC. Table 5 shows the relative fit of the two models. As can be seen, the log-likelihood was smaller for the ABDM than for the Q-diffusion model. AIC preferred the Q-diffusion model over the ABDM, whereas BIC preferred the ABDM over the Q-diffusion model. This difference between the two criteria is due to the higher penalty for model complexity imposed by BIC, which aims to select simpler models at small sample sizes. These results thus suggest that the ABDM provided a good account of the data at such a small sample size, despite being much simpler than the Q-diffusion model.

**Table 5:** Relative model fits.

	AIC	BIC	Log-likelihood	No. of parameters
ABDM	3,812.9	4,366.5	−1,792.5	114
Q-Diffusion	3,377.3	4,455.4	−1,466.7	222

**Table 6:** Relative model fits.

	AIC	BIC	Log-likelihood	No. of parameters
ABDM	8,798.3	10,173.8	−4,170.1	229
Q-Diffusion	8,341.4	11,026.3	−3,723.7	447

We used fivefold cross-validation to assess the absolute model fit. Firstly, we divided the data into five folds of 19 persons each, removed one fold from the data and fitted both models to the remaining data (person-based fit). Secondly, we divided the data into five folds of two items each, removed one fold from the data and fitted both models to the remaining data (item-based fit). We used the results of the person-based fit to compute the item parameters for the item fold removed in the item-based fit, and we used the results of the item-based fit to compute the person parameters for the person fold removed in the person-based fit. We subsequently combined the person and item parameters obtained in the two steps and used expressions (2) and (3) to predict RTs and accuracies for the person-by-item combinations contained in the removed folds. This was repeated for all 25 person-by-item folds.

Figure 11 shows the deviation between observed and predicted RTs (top row of plots) and accuracies (bottom row of plots) for the ABDM (left column of plots) and the Q-diffusion model (right column of plots). Each column of dots shows the results for the 19 persons in one person fold, each cluster of five columns of dots shows the results for all five person folds on one item, and each group of two items shows the results for all five person folds on one item fold. As can be seen, the ABDM tended to slightly overpredict RTs and produced more extreme outliers than the Q-diffusion model. For the Q-diffusion model, prediction errors clustered symmetrically around 0 with fewer outliers.

The results for the predicted accuracy show the opposite pattern of the response times. For the ABDM, prediction errors clustered closely around 0 with a few negative outliers. For the Q-diffusion model, prediction errors were larger and the model tended to underpredict accuracy.

Taken together, the comparison of the relative fits was ambiguous in that some criteria preferred the ABDM, whilst others preferred the hierarchical model or the Q-diffusion model. In terms of absolute fit, the ABDM tended to capture the accuracy data better than its competitor models, whilst the hierarchical model and the Q-diffusion model provided a better account for the RT data. A possible explanation for the worse performance of the ABDM is the very small number of items, which might have compromised the estimation of the person parameters. This explanation is also supported by the fact that the posterior predictive ranges for the person RTs and accuracies in Fig. 10 are much wider than the posterior predictive ranges for the item RTs and accuracies.

## Appendix D: Further Cross-Validation Results for the comparison of the ABDM and the Q-Diffusion Model

The comparison of the relative model fit by means of AIC and BIC is shown in Table 6. As reported in the main text, the log-likelihood was smaller for the ABDM than for the Q-diffusion model. Whilst AIC preferred the Q-diffusion model over the ABDM, BIC preferred the ABDM over the Q-diffusion model. This difference between the two criteria is due to the higher penalty for model complexity imposed by BIC.

The comparison of the absolute fit is presented in Fig. 12. As can be seen, the prediction errors for RTs for the ABDM clustered symmetrically around 0, with a few large positive outliers. This means that the ABDM predicted RTs were largely unbiased, except for very long RTs, in which case the ABDM tended to underpredict observed RTs. For the Q-diffusion model, prediction errors also clustered symmetrically around 0. The spread of the prediction error for the Q-diffusion model was comparable to that for the ABDM. Finally, the Q-diffusion model showed a similar tendency to underpredict long RTs as the ABDM.

The results for the predicted accuracy are again similar between the two models. For the ABDM, prediction errors tend to be positive, which indicates a tendency to underpredict accuracies. Nevertheless, most prediction errors are close to 0, which means that the ABDM generally captured the probability of a correct response well. The Q-diffusion model showed a similar tendency to underpredict accuracies but, in general, predicted the probability of a correct response well, with most prediction errors being slightly larger than 0. The spread of the prediction errors is also comparable to those observed for the ABDM. Taken together, both models provided a comparable absolute fit to the data.

## Footnotes

1 Although DIC should preferably be computed based on the integrated likelihood (Celeux et al., 2006), integrating the likelihood of the models under consideration here requires additional simplifying assumptions and might still yield normalising constants that are not available in closed form (Marsman et al., 2019).

2 As we estimated the boundary-to-volatility ratio for the ABDM before obtaining posterior samples, DIC neglects the influence of this parameter on model fit.

## References

[CR1] Allan Cheyne J, Solman GJ, Carriere JS, Smilek D (2009). Anatomy of an error: A bidirectional state model of task engagement/disengagement and attention-related errors. Cognition.

[CR2] Anderson TW (1960). A modification of the sequential probability ratio test to reduce the sample size. The Annals of Mathematical Statistics.

[CR3] Birnbaum, A. (1968). Some latent trait models and their use in inferring an examinee’s ability. In F. M. Lord & M. R. Novick (Eds.), *Statistical theories of mental test scores* (pp. 397–479). Reading, MA: Addison-Wesley.

[CR4] Borst G, Kievit RA, Thompson WL, Kosslyn SM (2011). Mental rotation is not easily cognitively penetrable. Journal of Cognitive Psychology.

[CR5] Britten KH, Shadlen MN, Newsome WT, Movshon AJ (1992). The analysis of visual motion: A comparison of neuronal and psychophysical performance. Journal of Neuroscience.

[CR6] Brown SD, Heathcote A (2008). The simplest complete model of choice response time: linear ballistic accumulation. Cognitive Psychology.

[CR7] Celeux G, Forbesy F, Robertz CP, Titteringtonx DM (2006). Deviance information criteria for missing data models. Bayesian Analysis.

[CR8] Cisek P, Puskas GA, El-Murr S (2009). Decisions in changing conditions: The urgencygating model. Journal of Neuroscience.

[CR9] Cox DR, Miller HD (1970). The theory of stochastic processes.

[CR10] Deneve, S. (2012). Making decisions with unknown sensory reliability. *Frontiers in Neuroscience,**6,*. 10.3389/fnins.2012.00075. 10.3389/fnins.2012.00075 PMC3367295 22679418

[CR11] Ditterich J (2006). Evidence for time-variant decision making. The European Journal of Neuroscience.

[CR12] Donkin C, Brown SD, Heathcote A (2009). The overconstraint of response time models: Rethinking the scaling problem. Psychonomic Bulletin & Review.

[CR13] Drugowitsch J, Moreno-Bote R, Churchland AK, Shadlen MN, Pouget A (2012). The cost of accumulating evidence in perceptual decision making. Journal of Neuroscience.

[CR14] Fox J-P (2006). Fixed effects IRT model. Behaviormetrika.

[CR15] Fox J-P, Glas CAW (2001). Bayesian estimation of a multilevel IRT model using Gibbs sampling. Psychometrika.

[CR16] Fox, J.-P., Klotzke, K., & Klein Entink, R. (2019). LogNormal Response Time Item Response Theory Models (R package version 0.4.0) [Computer software]. Retrieved from https://cran.r-project.org/web/packages/LNIRT/index.html.

[CR17] Gelfand AE, Smith AF (1990). Sampling-based approaches to calculating marginal densities. Journal of the American Statistical Association.

[CR18] Gelman A, Carlin JB, Stern HS, Dunson DB, Vehtari A, Rubin DB (2013). Bayesian Data Analysis.

[CR19] Gelman A, Rubin DB (1992). Inference from iterative simulation using multiple sequences. Statistical Science.

[CR20] Haladyna TM (2004). Developing and validating multiple-choice test items.

[CR21] Hanks TD, Mazurek ME, Kiani R, Hopp E, Shadlen MN (2011). Elapsed decision time affects the weighting of prior probability in a perceptual decision task. Journal of Neuroscience.

[CR22] Hawkins GE, Mittner M, Boekel W, Heathcote A, Forstmann BU (2015). Toward a model-based cognitive neuroscience of mind wandering. Neuroscience.

[CR23] Heathcote, A., & Love, J. (2012). Linear deterministic accumulator models of simple choice. *Frontiers in Psychology,**3,*. 10.3389/fpsyg.2012.00292. 10.3389/fpsyg.2012.00292 PMC3425963 22936920

[CR24] Kiefer J (1953). Sequential minimax search for a maximum. Proceedings of the American Mathematical Society.

[CR25] Laming DRJ (1973). Mathematical psychology.

[CR26] Lerche V, Voss A (2019). Experimental validation of the diffusion model based on a slow response time paradigm. Psychological Research.

[CR27] Marsman M, Sigurdardóttir H, Bolsinova M, Maris G (2019). Characterizing the manifest probability distributions of three latent trait models for accuracy and response time. Psychometrika.

[CR28] Matzke D, Wagenmakers E-J (2009). Psychological interpretation of the ex-Gaussian and shifted Wald parameters: A diffusion model analysis. Psychonomic Bulletin & Review.

[CR29] Mersmann, O., Trautmann, H., Steuer, D., & Bornkamp, B. (2018). truncnorm: Truncated normal distribution (R package version 1.0-8) [Computer software]. Retrieved from https://cran.rproject.org/web/packages/truncnorm/index.html.

[CR30] Mislevy RJ (1985). Estimation of latent group effects. Journal of the American Statistical Association.

[CR31] Molenaar, D. (2015). diffIRT (R package version 1.5) [Computer software]. Retrieved from https://cran.r-project.org/web/packages/diffIRT/index.html.

[CR32] Molenaar D, Tuerlinckx F, van der Maas HLJ (2015). Fitting diffusion item response theory models for responses and response times using the R package diffIRT. Journal of Statistical Software.

[CR33] Mooneyham BW, Schooler JW (2013). The costs and benefits of mind-wandering: A review. Canadian Journal of Experimental Psychology.

[CR34] Mrazek MD, Smallwood J, Franklin MS, Chin JM, Baird B, Schooler JW (2012). The role of mind-wandering in measurements of general aptitude. Journal of Experimental Psychology: General.

[CR35] Osterlind SJ (1998). What is constructing test items?.

[CR36] Palmer J, Huk AC, Shadlen MN (2005). The effect of stimulus strength on the speed and accuracy of a perceptual decision. Journal of Vision.

[CR37] Ranger J, Kuhn J (2018). Modeling responses and response times in rating scales with the linear ballistic accumulator. Methodology.

[CR38] Ratcliff R (1978). A theory of memory retrieval. Psychological Review.

[CR39] Ratcliff R (2008). The EZ-diffusion method: Too EZ?. Psychonomic Bulletin & Review.

[CR40] Ratcliff R, Frank MJ (2012). Reinforcement-based decision making in corticostriatal circuits: Mutual constraints by neurocomputational and diffusion models. Neural Computation.

[CR41] Ratcliff R, McKoon G (2008). The Diffusion Decision Model: theory and data for two-choice decision tasks. Neural Computation.

[CR42] Ratcliff R, Smith PL, Brown SD, McKoon G (2016). Diffusion Decision Model: Current issues and history. Trends in Cognitive Sciences.

[CR43] Ratcliff R, Thapar A, Gomez P, McKoon G (2004). A diffusion model analysis of the effects of aging in the lexical decision task. Psychology and Aging.

[CR44] Rouder JN, Province JM, Morey RD, Gomez P, Heathcote A (2015). The lognormal race: A cognitive-process model of choice and latency with desirable psychometric properties. Psychometrika.

[CR45] Schilling RL, Partzsch L (2014). Brownian motion.

[CR46] Schooler, J. W., Mrazek, M. D., Franklin, M. S., Baird, B., Mooneyham, B. W., Zedelius, C., & Broadway, J. M. (2014). The middle way: Finding the balance between mindfulness and mind-wandering. In B. H. Ross (Ed.), Psychology of learning and motivation (Chap. 1, Vol. 60, pp. 1–33). Academic Press.

[CR47] Shadlen MN, Kiani R (2013). Decision making as a window on cognition. Neuron.

[CR48] Singmann, H., Scott, B., Gretton, M., Heathcote, A., Voss, A., Voss, J., & Terry, A. (2016). rtdists: Response time distributions (R package version 0.6-6) [Computer software]. Retrieved from https://cran.r-project.org/web/packages/rtdists/index.html.

[CR49] Stone M (1960). Models for choice-reaction time. Psychometrika.

[CR50] Thura D, Beauregard-Racine J, Fradet C-W, Cisek P (2012). Decision making by urgency gating: Theory and experimental support. Journal of Neurophysiology.

[CR51] Thura D, Cisek P (2014). Deliberation and commitment in the premotor and primary motor cortex during dynamic decision making. Neuron.

[CR52] Thura D, Cos I, Trung J, Cisek P (2014). Context-dependent urgency influences speedaccuracy trade-offs in decision-making and movement execution. Journal of Neuroscience.

[CR53] Tuerlinckx F, De Boeck P (2005). Two interpretations of the discrimination parameter. Psychometrika.

[CR54] Van der Linden WJ (2009). Conceptual issues in response-time modeling. Journal of Educational Measurement.

[CR55] Van der Maas HLJ, Molenaar D, Maris G, Kievit RA, Borsboom D (2011). Cognitive psychology meets psychometric theory: On the relation between process models for decision making and latent variable models for individual differences. Psychological Review.

[CR56] Van Rijn PW, Ali US (2017). A comparison of item response models for accuracy and speed of item responses with applications to adaptive testing. British Journal of Mathematical and Statistical Psychology.

[CR57] Vandekerckhove J, Verheyen S, Tuerlinckx F (2010). A crossed random effects diffusion model for speeded semantic categorization decisions. Acta Psychologica.

[CR58] Voss A, Rothermund K, Voss J (2004). Interpreting the parameters of the diffusion model: An empirical validation. Memory and Cognition.

[CR59] Wagenmakers EJ, Van der Maas HLJ, Grasman RPPP (2007). An EZ-diffusion model for response time and accuracy. Psychonomic Bulletin & Review.

[CR60] Wagenmakers E-J, Ratcliff R, Gomez P, McKoon G (2008). A diffusion model account of criterion shifts in the lexical decision task. Journal of Memory and Language.

[CR61] Wald A, Wolfowitz J (1948). Optimum character of the sequential probability ratio test. The Annals of Mathematical Statistics.

[CR62] Zwinderman AH (1991). A generalized Rasch model for manifest predictors. Psychometrika.

